# Addition of *Aegilops* U and M Chromosomes Affects Protein and Dietary Fiber Content of Wholemeal Wheat Flour

**DOI:** 10.3389/fpls.2017.01529

**Published:** 2017-09-06

**Authors:** Marianna Rakszegi, István Molnár, Alison Lovegrove, Éva Darkó, András Farkas, László Láng, Zoltán Bedő, Jaroslav Doležel, Márta Molnár-Láng, Peter Shewry

**Affiliations:** ^1^Agricultural Institute, Centre for Agricultural Research, Hungarian Academy of Sciences Martonvásár, Hungary; ^2^Department of Plant Science, Rothamsted Research Harpenden, United Kingdom; ^3^Institute of Experimental Botany, Centre of the Region Haná for Biotechnological and Agricultural Research Olomouc, Czechia

**Keywords:** wheat, *Aegilops*, dietary fiber, β-glucan, arabinoxylan, U and M genomes

## Abstract

Cereal grain fiber is an important health-promoting component in the human diet. One option to improve dietary fiber content and composition in wheat is to introduce genes from its wild relatives *Aegilops biuncialis* and *Aegilops geniculata*. This study showed that the addition of chromosomes 2U^g^, 4U^g^, 5U^g^, 7U^g^, 2M^g^, 5M^g^, and 7M^g^ of *Ae. geniculata* and 3U^b^, 2M^b^, 3M^b^, and 7M^b^ of *Ae. biuncialis* into bread wheat increased the seed protein content. Chromosomes 1U^g^ and 1M^g^ increased the proportion of polymeric glutenin proteins, while the addition of chromosomes 1U^b^ and 6U^b^ led to its decrease. Both *Aegilops* species had higher proportions of β-glucan compared to arabinoxylan (AX) than wheat lines, and elevated β-glucan content was also observed in wheat chromosome addition lines 5U, 7U, and 7M. The AX content in wheat was increased by the addition of chromosomes 5U^g^, 7U^g^, and 1U^b^ while water-soluble AX was increased by the addition of chromosomes 5U, 5M, and 7M, and to a lesser extent by chromosomes 3, 4, 6U^g^, and 2M^b^. Chromosomes 5U^g^ and 7M^b^ also affected the structure of wheat AX, as shown by the pattern of oligosaccharides released by digestion with endoxylanase. These results will help to map genomic regions responsible for edible fiber content in *Aegilops* and will contribute to the efficient transfer of wild alleles in introgression breeding programs to obtain wheat varieties with improved health benefits.

**Key Message:** Addition of *Aegilops* U- and M-genome chromosomes 5 and 7 improves seed protein and fiber content and composition in wheat.

## Introduction

Because of its central role in the human diet, wheat is one of the major sources of dietary fiber (DF). The major DF components in wheat grain are the cell wall polysaccharides, arabinoxylan (AX) and (1-3)(1-4)-β-D-glucan (β-glucan), which account for about 70 and 20%, respectively, of the total cell wall polysaccharides in the starchy endosperm (and hence white flour) (Mares and Stone, 1973). AX and β-glucan occur in soluble and insoluble forms, which may differ in their health benefits. Insoluble DF lowers transit time and increases fecal bulk, defecation frequency, and the binding of carcinogens, while soluble DF reduces the risk of coronary heart disease and type II diabetes. DF components, in particular AX, also affect the processing properties of wheat, with respect to breadmaking, gluten-starch separation, the quality for livestock feed and fermentation to produce alcohol for beverages and biofuel (Courtin and Delcour, 2002; Frederix et al., 2004; Shewry et al., 2010b).

The content and composition of DF polysaccharides varies among cereal species. While wheat and rye are rich in AX, barley and oat have high β-glucan content. AX, the main pentosan component of the wheat grain, has a backbone chain of β-D-xylopyranosyl (Xylp) residues linked through (1-4)-glycosidic linkages. Some of the Xylp residues are monosubstituted with α-L-arabinofuranosyl (Araf) residues at position 3, or disubstituted at positions 2 and 3 of the same Xylp residues (Perlin, 1951; Renard et al., 1990; Hoffmann et al., 1991; Izydorczyk and Biliaderis, 1994). The AX in the secondary walls of the pericarp and seed coat tissues of the bran may also contain 4-*O*-methyl α-D-glucuronic acid as an additional substituent at position 2 of Xylp units (Schooneveld-Bergmans et al., 1999).

The optimum amount of AX to maintain good breadmaking quality while improving the health benefits in human diets will depend on several factors, including the molecular weight of the AX, the arabinose/xylose (A/X) ratio, the particle size of the fiber and the ferulic acid content (Morales-Ortega et al., 2013). Increased substitution of the xylopyranosyl residues with arabinofuranosyl residues is usually characterized by the ratio of the A/X present in the AX molecule (Ordaz-Ortiz and Saulnier, 2005), with a higher A/X ratio being associated with higher substitution and higher molecular weight. However, a lower A/X ratio of TOT-AX is associated with lower substitutions, lower molecular weight, and better end-use quality. Biliaderis et al. (1995) and Courtin and Delcour (1998) reported that high molecular weight (HMW) AX had greater effects on water absorption and on development time than lower molecular weight WE-AX. A lower amount of HMW polymer reduces the negative effects of fiber on technological properties and breadmaking quality, but the ability of AX to form highly viscous solutions decreases (Buksa et al., 2016). WE-AX could be characterized by lower molecular weight (2–20 kDa) and a lower A/X ratio (0.5–0.6) than insoluble AX (100–120 kDa or 300–600 kDa, 0.3–1.1) (Saulnier et al., 2007), with less negative effects on the quality.

β-Glucan is particularly important as a DF component in barley and oats and our previous studies indicated that this was also true for *Aegilops* species (unpublished data). The (1–3,1–4)-β-D-glucans are linear, unbranched polymers in which the β-D-glucopyranosyl residues are joined by both (1–3) and (1–4) glucosidic linkages. Single (1–3) linkages are separated by two or more (1–4) linkages, and regions of two or three adjacent (1–4) linkages predominate. The distribution of oligosaccharides in β-glucan differs in different cereal species (Cui et al., 2000; Lazaridou et al., 2004), with the relative proportion of trisaccharide [DP3 (degree of polymerization)] decreasing from wheat (67–72%), to barley (52–69%), and oats (53–61%) and the relative amount of tetrasaccharide (DP4) following the opposite trend. Differences in the ratio of DP3:DP4 may also occur within the same cereal species, which may be attributed to genotypic and environmental factors (Miller et al., 1993; Jiang and Vasanthan, 2000; Storsley et al., 2003; Wood et al., 2003).

Differences in the linkage distribution and molecular weight of β-glucan are likely to affect its solubility and viscosity (Lazaridou and Biliaderis, 2007; Cui and Wood, 2000), which are considered to be key parameters determining health benefits (Wood, 2007). However, high viscosity conferred by β-glucan has negative effects on feed intake, feed conversation rate, and weight gain, and may result in sticky feces when used to feed chickens (Hesselman et al., 1981). In malt and beer production high viscosity causes problems with haze formation and wort filtration (Bamforth, 2010), and reduces yield in starch production. In contrast, high contents of soluble β-glucan are favored for food products as they may reduce serum cholesterol levels and regulate blood glucose level (McIntosh et al., 1991; Cavallero et al., 2002; Wood, 2007). From a processing point of view, HMW β-glucan results in higher water absorption and viscosity (Skendi et al., 2009), lower loaf volume and height (Symons and Brennan, 2004; Izydorczyk and Dexter, 2008; Skendi et al., 2009), and stiffer dough (Cleary et al., 2007) than LMW β-glucan. Therefore it is technologically easier to incorporate LMW barley β-glucan fractions into breads. The flow behavior and gelling properties of β-glucan can also vary with the concentration and molecular weight (Lazaridou et al., 2003; Vaikousi et al., 2004; Skendi et al., 2009).

DF has been studied widely in wheat and other cereals (rye, barley, spelt), primarily focusing on variability in the amount and composition (Saulnier et al., 2007; Andersson et al., 2008; Gebruers et al., 2008; Rakszegi et al., 2008; Shewry et al., 2008; Ward et al., 2008), genetic control (Cyran et al., 1996; Boros et al., 2002; Burton et al., 2006; Mitchell et al., 2007; Charmet et al., 2009; Doblin et al., 2010; Nemeth et al., 2010; Quraishi et al., 2011; Taketa et al., 2012), heritability (Martinant et al., 1999; Li et al., 2009; Gebruers et al., 2010; Shewry et al., 2010b,c), and effects on animal and human health (Bedford and Schulze, 1998; Brouns et al., 2013; Lafiandra et al., 2014; Pirgozliev et al., 2015) and food processing (Courtin and Delcour, 2002; Frederix et al., 2004; Bonnand-Ducasse et al., 2010; Noort et al., 2010; Shewry et al., 2010a; Jones et al., 2015; Heinio et al., 2016). The health benefits of DFs triggered a search for wild alleles suitable to increase the level of DFs in wheat, mainly in the genus *Triticum* (Marcotuli et al., 2015, 2016). However, to date these efforts have neglected wild relatives of wheat from the genus *Aegilops*, although these are important donors of new genes and alleles for wheat breeding.

The genus *Aegilops* is the closest relative of genus *Triticum* and consists of 11 diploid, 10 tetraploid, and 2 hexaploid species (van Slageren, 1994) with six different genomes (D, S, U, C, N, and M), indicating the great genetic diversity of the genus. Twelve *Aegilops* species contain the U and/or M genomes, and two of these, the allotetraploid *Aegilops biuncialis* (2n = 4x = 28, U^b^U^b^M^b^M^b^) and *Aegilops geniculata* (2n = 4x = 28, U^g^U^g^M^g^M^g^), evolved from hybridization between the diploid *Aegilops comosa* (2n = 2x = 14, MM) and *Aegilops umbellulata* (2n = 2x = 14, UU) (van Slageren, 1994). Because of their great ecological adaptability, these species are promising sources of genes providing resistance to diseases (*Lr9*, *Lr57*, *Sr34*, *Yr8*, *Yr40*, *Pm29*) and tolerance to abiotic stresses such as salt, drought, frost, and heat stress (Friebe et al., 1996; Rekika et al., 1997; Zaharieva et al., 2001a,b; Molnár et al., 2004; Colmer et al., 2006; Schneider et al., 2008; Dulai et al., 2014).

Besides providing stress tolerance, the U- and M-genomes of *Aegilops* species are also rich reservoirs of genes for improving the nutritional quality of the wheat grain. Bálint et al. (2001) compared the contents of micro- and macronutrients in the grain of diploid, tetraploid, and hexaploid *Triticum* and *Aegilops* species and found that the Cu, Zn, Ca, and Mg contents were significantly higher in *Ae. biuncialis* and *Ae. geniculata* than in wheat. Rawat et al. (2009) also reported two- to threefold higher contents of iron and zinc in *Ae. geniculata* grain than in bread and durum wheats. These results were confirmed by Farkas et al. (2014), who found 1.5- to 2-fold higher contents of K, Zn, Fe, and Mn in *Ae. biuncialis* than in bread wheat. In wheat, the major determinants of grain processing quality are the gluten storage proteins, which consist of monomeric gliadins (Gli) and polymeric glutenins (Glu), with the gliadins determining dough extensibility and the glutenins its elasticity. Good quality is associated with a high ratio of unextractable polymeric glutenin proteins (UPP; Shewry et al., 1986; Larroque and Békés, 2000) rich in HMW subunits of glutenin. Variation in the HMW subunit composition of *Ae. geniculata* was reported by Medouri et al. (2015), who identified a total of 27 alleles at the two HMW glutenin loci, *Glu-M1* and *Glu-U1*, resulting in 29 HMW glutenin protein patterns. Other studies on the composition of glutenins and gliadins in *Aegilops* species have also been reported (Bandou et al., 2009; Kozub et al., 2011; Wang et al., 2012, 2015; Ahmadpoor et al., 2014; Dai et al., 2015; Medouri et al., 2015; Garg et al., 2016).

While information is already available for micronutrients and gluten storage proteins of *Aegilops*, the content and composition of the grain DF components AX and β-glucan have not been reported yet in species with the U and M genomes.

One strategy to increase genetic variation in bread wheat is to introduce new genes by interspecific hybridization. Several useful agronomic traits have already been transferred from *Aegilops* into the wheat gene pool by developing wheat–*Aegilops* hybrids and chromosome addition and translocation lines, as reviewed by Schneider et al. (2008). Wheat-alien disomic chromosome addition lines are excellent genetic resources to study the performance of transferred alien traits in the wheat genetic background and to assign key genes to alien chromosomes. Wheat–*Ae. biuncialis* addition lines carrying chromosomes 1U^b^, 3U^b^, 2M^b^, 3M^b^, and 7M^b^ were developed by Molnár-Láng et al. (2002) and Schneider et al. (2005), while a complete set of wheat–*Ae. geniculata* addition lines was developed by Friebe et al. (1999).

The advent of next-generation sequencing technologies (Margulies et al., 2005) and improvements in the flow-cytometric sorting of mitotic chromosomes (Doležel et al., 2014; Rey et al., 2015) allow the identification of the gene repertoire of individual chromosomes and the development of gene-specific markers for large and complex Triticeae genomes, such as barley, rye, bread wheat (Mayer et al., 2011; Martis et al., 2013; The International Wheat Genome Sequencing Consortium [IWGSC], 2014), and their wild relatives (Tiwari et al., 2015). Molnár et al. (2016) reported the flow-sorting of the U- and M-genome chromosomes from the diploid progenitors of *Ae. biuncialis* and *Ae. geniculata*, *Ae. umbellulata* (UU), and *Ae. comosa* (MM). The high purity of the sorted fractions allowed the sequencing of the U genome based on the individual chromosomes of *Ae. umbellulata*, thereby producing genomic resources to identify the chromosomal positions in *Aegilops* of orthologs of the key genes responsible for agronomic traits of interest.

The main goals of the present study were to determine the effects of added *Ae. biuncialis* and *Ae. geniculata* chromosomes on the content and composition of the grain storage protein and DF components of hexaploid wheat, by carrying out detailed biochemical analyses of *Ae. biuncialis* and *Ae. geniculata* accessions and wheat–*Aegilops* addition lines. The chromosomal positions of putative orthologs of the key genes determining these components were also identified using *Ae. umbellulata* chromosome sequences.

## Materials and Methods

### Plant Material

Bread wheat (cv. Chinese Spring)/*Ae. geniculata* (TA2899) chromosome addition lines 1U^g^, 2U^g^, 3U^g^, 4U^g^, 5U^g^, 6U^g^, 7U^g^, 1M^g^, 2M^g^, 3M^g^, 5M^g^, 6M^g^, and 7M^g^ were kindly provided by Dr. Bernd Friebe (Kansas State University, Manhattan, KS, United States) and maintained by the Cereal Genebank, Martonvásár, while a set of bread wheat (line Mv9kr1)/*Ae. biuncialis* (MvGB642) chromosome addition lines 1U^b^, 1U^b^6U^b^, 3U^b^, 2M^b^, 3M^b^, and 7M^b^ were produced in Martonvásár (Molnár-Láng et al., 2002; Schneider et al., 2005). The recessive crossability allele *kr1* was transferred from bread wheat cv. Chinese Spring (CS) into bread wheat cv. Martonvásári 9 (Mv9) by backcrossing the Mv9 × CS hybrids with Mv9. The Mv9*kr1* line carries recessive crossability alleles *Kr1* and *Kr2*, but the genotype is 93.75% Mv9 (Molnár-Láng et al., 1996). *Ae. geniculata* or *Ae. biuncialis* accessions were provided by the Cereal Genebank, Martonvásár (MvGB), by the Wheat Genetics Resource Center, Kansas State University, United States (TA) and by the Institute of Plant Genetics and Crop Plant Research, Gatersleben, Germany (AE) and maintained by the Cereal Genebank, Martonvásár.

### Growing Conditions

#### Glasshouse Experiment

Seeds were germinated on wet filter paper in Petri dishes for 3 days at room temperature, and then potted into Jiffy7 pellets^1^. The 5-day-old seedlings were vernalized at 4°C for 6 weeks under low light intensity (20 μmol m^-2^ s^-1^). After vernalization, seedlings were grown in individual pots (one plant/2 l pot) filled with a 3:2:1 mixture of garden soil, compost, and sand, and placed randomly in the greenhouse (Global Glasshouse Venlo) for 12 weeks. Each pot was fertilized every 10 days for four times from the second week after planting with 150 ml of 0.1% g/v % complex fertilizer containing 14% N, 7% P_2_O_5_, 21% K_2_O, 1% Mg, and 1% micronutrients including B, Cu, Mn, Fe, Zn (Volldünger Classic, Gartenhilfe GmbH., Austria). Growth conditions were as follows: the initial 11/7°C day/night temperature and 13 h photoperiod gradually increased to 23/17 °C day/night temperature and 16 h photoperiod (16 h light/8 h dark) at maturity (12 weeks). The seeds of 10 plants per genotype were used for analysis.

#### Field Trial

The *Aegilops* accessions, the wheat–*Ae. geniculata* and wheat–*Ae. biuncialis* addition lines together with their parental lines were grown in chernozem soil in the experimental field in Martonvásár (2012/2013) characterized by low-input conditions (no fertilizers, herbicides, insecticides, and fungicides applied during the growing seasons). The weather conditions during the growing period (basically between October and July) could be characterized by the total precipitation of 387.5 mm and average temperature of 8.0°C (Mikó et al., 2014).

Each genotype was sown in randomized complete block design with two replications on October 15, 2012. The plots consisted of 5 × 1 m rows with a row distance of 15 cm and 50 seeds per plot. Plots were hand harvested at maturity and grain was stored at 4°C.

### Methods

Four grams of seed from each sample was milled using a Retsch Mixer Mill MM 200 ball mill to produce wholemeal samples, which were immediately cooled and stored at -20°C until compositional analysis.

#### Thousand Kernel Weight

Thousand kernel weights (TKW) were determined by the standard MSZ 6367/4-86 (1987) method. Duplicate analyses were carried out on each sample.

#### Protein

Crude protein content was determined by the Kjeldahl method, consistent with International Association for Cereal Science and Technology ICC 105/2 (1995), using a Kjeltec 1035 Analyzer instrument. Duplicate analyses were carried out on each sample.

#### Protein Composition

Size exclusion-high-performance liquid chromatography was used to determine the glutenin, gliadin, and albumin + globulin contents and the UPP (UPP% = insoluble glutenin/soluble + insoluble glutenin) content using a modification of the Batey et al. (1991) method. Ten milligram flour was suspended in 1 ml 0.5% (w/v) SDS in phosphate buffer (pH 6.9) and sonicated for 15 s. After centrifugation, the supernatant was filtered on a 0.45 μm PVDF filter. Analyses were performed on a Phenomenex BIOSEP-SEC 4000 column in acetonitrile buffer [0.05% (v/v) trifluoroacetic acid and 0.05% (v/v) acetonitrile] with a running time of 10 min (2 ml/min flow rate). Proteins were detected by absorption at 214 nm.

#### Quantitative Determination of Total and Water-Extractable Pentosans

Total and water-extractable pentosans, in which AX is the main component, were determined using a colorimetric method, as described by Douglas (1981) and Finnie et al. (2006). A total of 12.5 ml Milli-Q water was added to 62.5 mg flour and shaken (TOT-pentosan). An aliquot of the suspension (0.5 ml) was diluted to 1.0 ml with water and 5 ml freshly prepared extraction solution was added [93.2% (v/v) acetic acid, 1.69% (v/v) HCl, 0.85% (w/v) phloroglucinol and 0.017% (w/v) glucose]. The tubes were placed in a boiling water bath for 25 min and the absorbance of the samples was measured after cooling at 552 and 510 nm. The remainder of the flour water suspension was shaken for 30 min to determine water-extractable pentosans (WE-pentosan). The suspension was centrifuged at 2500 *g* for 10 min, 0.5 ml of the supernatant was removed and diluted to 1.0 ml with water, and 5 ml of extraction solution was added. The sample was then boiled for 25 min and the absorbance was measured after cooling at 552 and 510 nm. The pentose concentration was determined by comparing the absorbance values with those of D-(+)-xylose standards. Duplicate analyses were carried out on each sample.

#### Quantitative Determination of β-Glucan

The total amount of mixed-linkage β-glucan was determined in wholemeal samples using a Megazyme kit (Megazyme, Bray, Ireland) (AACC International, 1995; International Association for Cereal Science and Technology ICC 166, 1998). Duplicate analyses were carried out on each sample.

#### Enzyme Fingerprinting of AX and β-Glucan

The protocol was adapted from Ordaz-Ortiz et al. (2004, 2005). One milliliter of 80% (v/v) ethanol was added to 100 mg of flour and heated in a 95°C water bath for 5–10 min to inactivate the enzymes. After centrifugation, the residue was washed first with 80% (v/v) ethanol and then with 95% (v/v) ethanol and dried using a Speedvac centrifugal evaporator. The dried powder was resuspended in 1 ml of water containing 16U of endoxylanase (Megazyme, *Trichoderma viride*, Xylanase M1, Bray, Ireland) and 2U of lichenase (Megazyme) and incubated at 40°C for 16 h with continuous rotation. After centrifugation, 0.6 ml of the supernatant was heated for 10 min in a 95°C hot water bath to inactivate the enzymes. The samples were then centrifuged and filtered using 0.45 μm Millex-HV syringe-driven filters. After water dilution (1:20), samples were injected onto an HPAEC system (high-performance anion-exchange chromatography) using a Carbopac PA1 analytical column (4 mm × 250 mm) (Ordaz-Ortiz et al., 2004, 2005). Duplicate analyses were carried out on each sample. The proportions of unsubstituted, monosubstituted, and disubstituted xylose residues in the AX oligosaccharides (AXOS) and the ratio of the DP3 and DP4 units of β-glucan are calculated from the peak areas (Saulnier et al., 2009; Toole et al., 2010), calculated as

monosubstituted (M) = (XA^3^XX) + 2(XA^3^A^3^XX) + 2(XA^3^XA^3^XX) + (XA^3^A^2+3^X) + (XA^3^XA^2+3^XX), disubstituted (D) = (XA^2+3^XX) + (XA^3^A^2+3^X) + (XA^3^XA^2+3^XX), unsubstituted (US) = X + XX + XXX, and total (TOT) = X + XX + XXX + XA^3^XX + XA^3^A^3^XX + XA^3^XA^3^XX + (XA^2+3^XX) + (XA^3^A^2+3^XX) + (XA^3^XA^2+3^XX) AXOS (**Supplementary Figure S1**).

#### DNA Sequence Analysis

Mitotic chromosomes 1U, 2US, 2UL, 3U, 4U, 5U, 6U, 7U, and 7UL of *Ae. umbellulata* (2n = 2x = 14, UU) accession AE740/03 were purified by flow sorting as described by Molnár et al. (2016). DNA was amplified from three samples of each chromosome using the Illustra GenomiPhi V2 DNA Amplification Kit (Šimková et al., 2008), pooled and sequenced with HiSeq 2000 (Illumina, Inc., San Diego, United States) using standard protocols. The DNA of each chromosome was sequenced on one lane of the instrument, representing nine lanes with ∼130 millions of paired-end reads (∼26 Gb) for each dataset. *De novo* assembly was done with a MaSuRCA assembler (Zimin et al., 2013) and contigs shorter than 200 bp were removed. The databases of Illumina reads and assembled contigs were made publicly available on the web site of IEB, Olomouc^2^. Sequences of key genes and alleles determining the composition and quantity of storage proteins, β-glucan, and AX in the grain were downloaded from a publicly available database^3^^,^^4^ (Schreiber et al., 2014) and used as queries for BLASTn searches against *Ae. umbellulata* chromosome sequences (Supplementary Tables 1–3). The first best hits with at least 75% sequence identity and a minimal alignment length of 200 bp were considered significant and used to obtain the chromosomal positions of key genes in the U genome of *Ae. umbellulata*.

#### Statistical Analyses

Two replications were made for the TKW, protein, β-glucan, pentosan, and AX measurements and if the difference between the two replicate samples was higher than 10% the measurement was repeated with two more replications. Three replicate samples were measured for Glu/Gli and UPP%. Least significant difference values between the addition lines, together with the parental wheat and *Aegilops* genotypes, were calculated at the *p* = 0.05 probability level using the Microsoft Excel program.

## Results

### Variation in Composition within Species, *Ae. biuncialis* and *Ae. geniculata*

**FIGURE 1:**
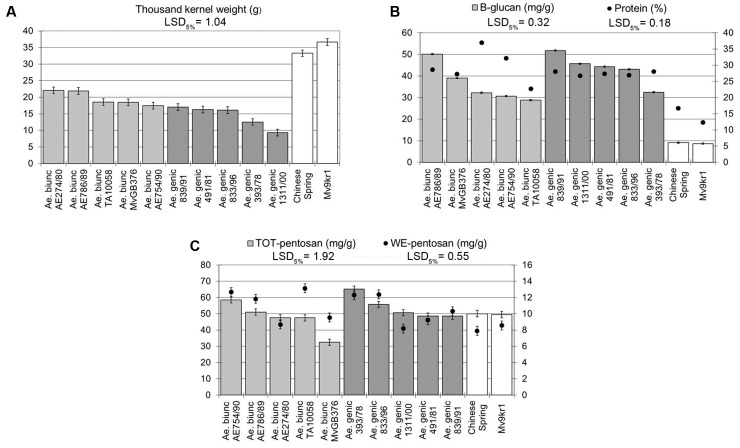
Thousand kernel weight and compositional properties of five *Aegilops biuncialis* and five *Aegilops geniculata* genebank accessions. **(A)** Thousand kernel weight, **(B)** protein and β-glucan content, **(C)** TOT-pentosan and WE-pentosan content. LSD, least significant difference; TOT, total; WE, water extractable.

No studies have yet been reported on the composition of DF in *Aegilops* species with the U and M genomes, but previous results on HMW glutenin subunit alleles showed wide variation within these species (Ahmadpoor et al., 2014; Dai et al., 2015; Medouri et al., 2015; Wang et al., 2015; Garg et al., 2016). In order to determine the extent of variation in the amounts of protein, β-glucan, TOT-pentosan, and WE-pentosan in *Ae. geniculata* and *Ae. biuncialis*, five genebank accessions of each species were studied. The *Ae. geniculata* and *Ae. biuncialis* accessions had significantly lower TKW than the two wheat genotypes (cv. Chinese Spring and line Mv9kr1) used in the present study (**Figure 1A**), with values ranging between 17.4 and 22.1 g (accessions AE754/90 and AE274/80, respectively) in *Ae. biuncialis*, and between 9.3 and 17.0 g (accessions AE1311/00 and AE839/91, respectively) in *Ae. geniculata*.

The β-glucan and protein content were significantly higher in all *Aegilops* accessions than in the wheat genotypes (**Figure 1B**). Within *Ae. biuncialis* and *Ae. geniculata*, the β-glucan content varied between ∼30 and 50 mg/g irrespective of the TKW of the accessions. Interestingly, the protein content was more variable among the *Ae. biuncialis* accessions (∼22–37%) than in *Ae. geniculata* genotypes (∼26%), where differences were not observed for this parameter. The amounts of TOT-pentosan and WE-pentosan also varied (∼30–60 and ∼8–14 mg/g, respectively), but with the exception of one accession (MvGB376) were similar to or above those in wheat (**Figure 1C**).

### Effect of *Aegilops* Chromosomes on Thousand Kernel Weight and Storage Protein Content in Wheat

**FIGURE 2:**
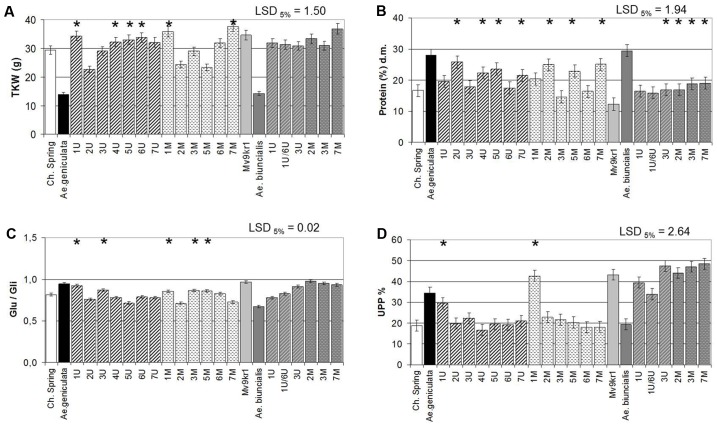
Compositional properties of mature grains of two lines of bread wheat (cv. Chinese Spring and Mv9kr1 line), two *Aegilops* species (*Ae. geniculata* and *Ae. biuncialis*), and wheat–*Aegilops* chromosome addition lines. **(A)** TKW, **(B)** protein, **(C)** Glu/Gli, **(D)** UPP%. Gli, gliadin; Glu, glutenin; LSD, least significant difference; TKW, thousand kernel weight; UPP, unextractable polymeric protein. ^∗^Significantly higher than the wheat (*Triticum aestivum*) control.

The parental *Ae. geniculata* TA2899 and *Ae. biuncialis* MvGB642 genotypes have significantly lower (<50%) TKW than the parental wheat genotypes cv. Chinese Spring and cv. Mv9kr1 (**Figure 2A**). In the Chinese Spring × *Ae. geniculata* combination, chromosome addition lines 1U^g^, 4-5-6U^g^, 1M^g^, and 7M^g^ showed significantly higher TKW than the wheat parent, while addition lines containing chromosomes 2U^g^, 2M^g^, and 5M^g^ exhibited significantly lower TKW. *Ae. biuncialis* chromosomes had no effect on this parameter. In parallel with the lower TKW values, the *Aegilops* accessions had higher protein content relative to wheat (**Figure 2B**), which can be attributed to the “yield dilution” effect, i.e., the high grain weight of wheat, resulting from the increased starch content and the consequent relative decrease in grain storage proteins. The addition of chromosomes 2U^g^, 4U^g^, 5U^g^, 7U^g^, 2M^g^, 5M^g^, and 7M^g^ of *Ae. geniculata* significantly increased the protein content of wheat cv. Chinese Spring, while the addition of *Ae. biuncialis* chromosomes 3U^b^, 2M^b^, 3M^b^, and 7M^b^ significantly increased that of wheat line Mv9kr1 (**Figure 2B**).

Under field conditions, the 6U^g^, 3M^g^, 6M^g^, and 7M^b^ additions exhibited the most stable TKW similar to wheat, while the TKW of all the other lines were decreased by adding the *Aegilops* chromosomes (**Supplementary Figure S2a**). It is probable that the very low TKW resulted the significantly higher protein contents of *Aegilops* than wheat (with the exception of 2M^b^ and 3M^b^), with 1U^b^, 1U/6U^b^, 2U^g^, 3U, 4U^g^, 2M^g^, and both 7M having the greatest effects (**Supplementary Figure S2b**).

The good processing quality of wheat is related to the high content of polymeric glutenins relative to gliadins (Glu/Gli) and to the high ratio of UPP (UPP%). *Ae. geniculata* accession TA2899 had a higher Glu/Gli ratio and UPP% than cv. Chinese Spring, whereas these two parameters were lower in *Ae. biuncialis* MvGB642 than in Mv9kr1 (**Figures 2C,D**). It should be noted that the model wheat genotype cv. Chinese Spring, which has poor processing quality properties, exhibited lower Glu/Gli ratio and UPP% than line Mv9kr1 whose breadmaking quality parameters are good. This is why the effect of *Aegilops* chromosomes on the quality parameters of wheat was more pronounced in cv. Chinese Spring than in Mv9kr1. In agreement with this, the added chromosomes 1U^g^ and 1M^g^ significantly increased the proportion of polymeric glutenin proteins (higher Glu/Gli ratio and UPP%) in the Chinese Spring background, which can be expected to result in improved processing quality (**Figures 2C,D**). The addition of chromosomes 3U^g^, 3M^g^, and 5M^g^ also increased the Glu/Gli ratio, but did not affect the UPP%. In the case of *Ae. biuncialis*, chromosomes 1U^b^ and 1U^b^/6U^b^ again had the greatest effects on the protein composition and the proportion of glutenin polymers (**Figures 2C,D**), but these were negative, with the proportion of monomeric gliadins increasing instead of the glutenins.

### Effect of *Aegilops* Chromosomes on β-Glucan and AX Content

**FIGURE 3:**
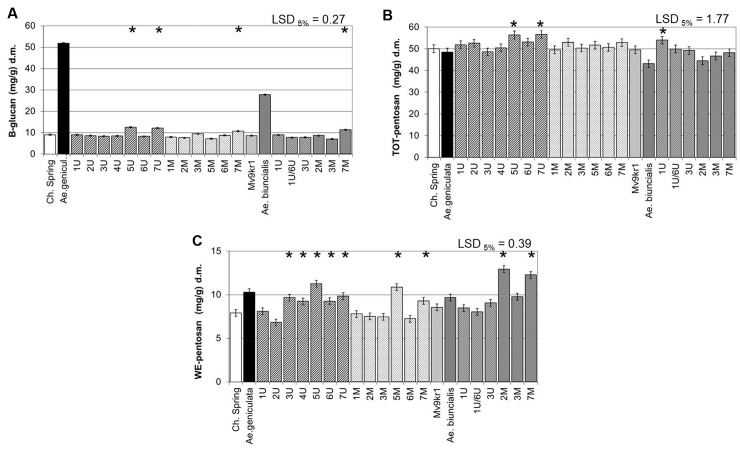
Compositional properties of mature grains of two lines of bread wheat (cv. Chinese Spring and Mv9kr1 line), two *Aegilops* species (*Ae. geniculata* and *Ae. biuncialis*), and wheat–*Aegilops* chromosome addition lines. **(A)** β-Glucan, **(B)** TOT-pentosan, and **(C)** WE-pentosan content. LSD, least significant difference; TOT, total; WE, water-extractable. ^∗^Significantly higher than the wheat (*T. aestivum*) control.

The β-glucan content expressed in mg/g dry weight of wholemeal (measured using the commercial kit for total β-glucan content) was about fivefold higher in *Ae. geniculata* TA2899 than in cv. Chinese Spring and 2.5-fold higher in *Ae. biuncialis* MvGB642 than in line Mv9kr1 (**Figure 3A**). Chromosome addition lines 5U^g^, 7U^g^, and 7M^g^, which had similar or higher TKW than wheat, had a positive effect on β-glucan content in the Chinese Spring genetic background. In the case of Mv9kr1 × *Ae. biuncialis* addition lines, significantly higher β-glucan content was also observed in the 7M^b^ addition line.

*Aegilops* chromosomes 5U^g^, 7U^g^, 7M^g^, and 7M^b^ were able to increase the β-glucan content of wheat wholemeal under field conditions, which fully support the results of the glass house experiments (**Supplementary Figure S2c**). Moreover, the significant effect of chromosome 1U^b^ was also shown in the field experiment.

The content of total AX (measured as TOT-pentosan) was similar in *Ae. geniculata* and cv. Chinese Spring and lower in *Ae. biuncialis* than in line Mv9kr1 (**Figure 3B**). The effects of chromosome additions were generally not significant, but small increases were observed with the addition of chromosomes 5U^g^ and 7U^g^, which increased the total AX content of wheat (measured as TOT-pentosan in mg/g dry weight of wholemeal), while in the Mv9kr1 genetic background only chromosome 1U^b^ gave a higher value than the parental wheat genotype (**Figure 3B**). No significant differences were observed in the contents of TOT-pentosan in the parental lines and the addition lines in the field experiment (**Supplementary Figure S2d**).

The water-extractable fraction of total pentosans (WE-pentosan) was higher in the *Aegilops* parents in comparison with the corresponding wheat genotypes (**Figure 3C**). At the level of single chromosomes, significantly higher WE-pentosan content was observed in chromosome addition lines 3U^g^, 4U^g^, 5U^g^, 6U^g^, 7U^g^ and 5M^g^ and 7M^g^, the highest positive effect being exerted by the group five chromosomes of *Ae. geniculata*. In the case of *Ae. biuncialis*, chromosome addition lines 2M^b^ and 7M^b^ showed the highest level of WE-pentosan, exceeding those of all the other genotypes investigated (wheat–*Ae. geniculata* and wheat–*Ae. biuncialis* additions). The results of the glasshouse experiment were supported by the field experiment. Similar to the glasshouse experiments, chromosome additions 5U^g^ and 5M^g^ showed the highest WE-pentosan contents among the Chinese Spring–*Ae. geniculata* addition lines, although their values were not significantly different from the wheat parent, while the *Ae. biuncialis* 7M^b^ chromosome addition had significantly higher WE-pentosan content than the parental wheat Mv9kr1 (**Supplementary Figure S2e**).

**FIGURE 4:**
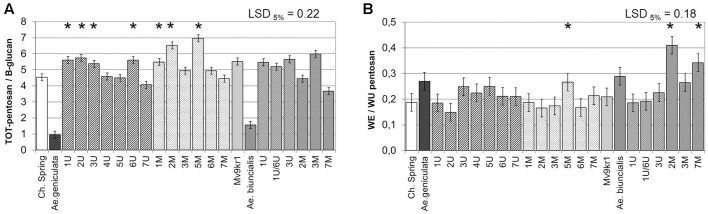
Quantitative ratio of TOT-pentosan to β-glucan **(A)** and WE to WU-pentosan **(B)** in mature grains of two lines of bread wheat (cv. Chinese Spring and Mv9kr1 line), two *Aegilops* species (*Ae. geniculata* and *Ae. biuncialis*), and wheat–*Aegilops* chromosome addition lines. LSD, least significant difference; TOT, total; WE, water extractable; WU, water-unextractable. ^∗^Significantly higher than the wheat (*T. aestivum*) control.

The ratio of TOT-pentosan (mainly AX) to β-glucan reflects the composition of non-starch cell wall polysaccharides. In the present experiment, the parental wheat genotypes had TOT-pentosan to β-glucan ratios of 4.5 and 5.5 (in cv. Chinese Spring and line Mv9kr1, respectively). In contrast, TOT-pentosan to β-glucan ratios of only 1.0 and 1.5 were determined for the *Ae. geniculata* TA2899 and *Ae. biuncialis* MvGB642 (**Figure 4A**), which was attributed to the high β-glucan content of the *Aegilops* genotypes (**Figure 3A**). In Chinese Spring–*Ae. geniculata* chromosome additions, this ratio was higher in lines containing chromosomes 1U^g^, 2U^g^, 3U^g^, 6U^g^, and 1M^g^, 2M^g^, and 5M^g^ than in the wheat parent, while the chromosome addition line 7U^g^ showed a lower value for this parameter. Within the set of *Ae. biuncialis* additions, chromosomes 2M^b^ and 7M^b^ significantly decreased the ratio of TOT-pentosan to β-glucan relative to wheat. The ratio of water-extractable to unextractable pentosans (WE/WU pentosan) indicates the relative amounts of the two pentosan fractions, which have different health-promoting effects. The higher WE/WU pentosan ratio showed that the two *Aegilops* accessions have higher proportions of water-extractable pentosans than the wheat parents (**Figure 4B**). A relatively higher amount of water-extractable pentosans was also detected in the wheat–*Aegilops* addition lines 5M^g^, 2M^b^, and 7M^b^ (**Figure 2C**).

### Effect of Added *Aegilops* Chromosomes on the Structure of β-Glucan and AX Polymers

**FIGURE 5:**
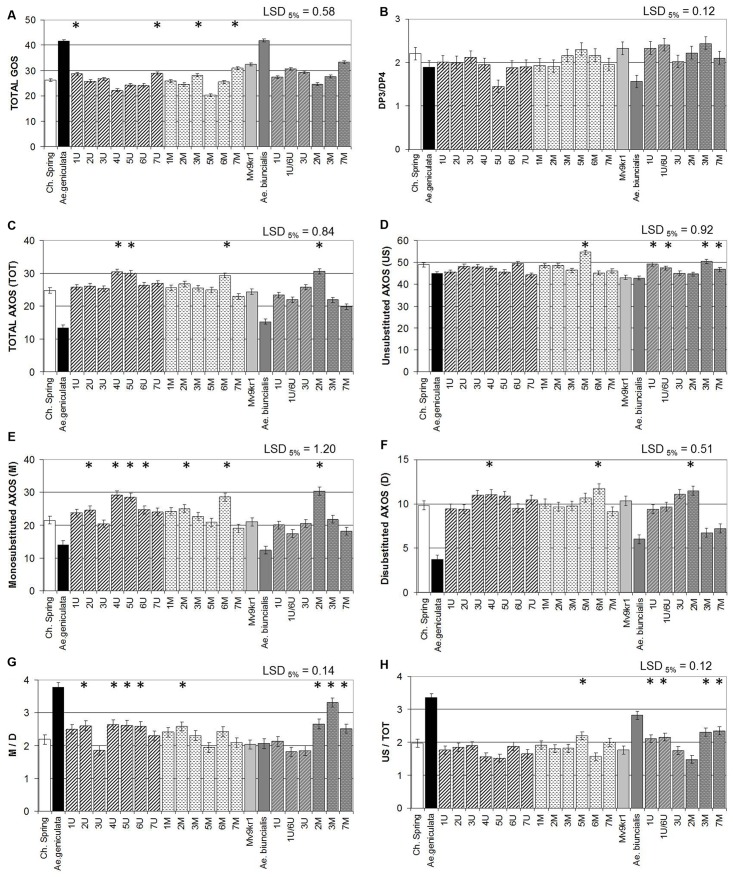
Quantity of arabinoxylan units in mature grains of two lines of bread wheat (cv. Chinese Spring and Mv9kr1 line), two *Aegilops* species (*Ae. geniculata* and *Ae. biuncialis*), and wheat–*Aegilops* chromosome addition lines after enzymatic fingerprinting. **(A)** Quantity of β-glucan units after enzymatic fingerprinting, **(B)** ratio of DP3 to DP4 units, **(C)** TOT-AXOS, **(D)** unsubstituted AXOS (US), **(E)** monosubstituted AXOS (M), **(F)** disubstituted AXOS (D), **(G)** M/D ratio, **(H)** US/TOT ratio. The amounts of unsubstituted, monosubstituted, and disubstituted xylose residues in the AXOS are derived from the AXOS peak areas, calculated as (M) = (XA^3^XX) + 2(XA^3^A^3^XX) + 2(XA^3^XA^3^XX) + (XA^3^A^2+3^X) + (XA^3^XA^2+3^XX), (D) = (XA^2+3^XX) + (XA^3^A^2+3^X) + (XA^3^XA^2+3^XX), (US) = X+XX+XXX, and (TOT) = X + XX + XXX + XA^3^XX + XA^3^A^3^XX + XA^3^XA^3^XX + (XA^2+3^XX) + (XA^3^A^2+3^XX) + (XA^3^XA^2+3^XX) AXOS, arabinoxylan oligosaccharide; DP, degree of polymerization; LSD, least significant difference; TOT, total. ^∗^Significantly higher than the wheat (*T. aestivum*) control.

Differences in the structure of β-glucan were determined after digestion with lichenase. Lichenase releases glucooligosaccharides (GOS) with degrees of polymerization (DP) of up to 10, with DP3 and DP4 GOS being the major forms. The values for TOTAL-GOS were between 1.6- and 1.3-fold higher in *Ae. geniculata* TA2899 and *Ae. biuncialis* MvGB642, respectively, than in wheat (**Figure 5A**). The ratio of DP3:DP4 glucan units, which represents the ratio of β-(1–3) to β-(1–4) bonds in the polymer, was significantly lower in both *Aegilops* species than in wheat (**Figure 5B**). The effect of chromosomes 7U^g^ and 7M^g^ on the TOTAL-GOS content, determined using HPAEC (**Figure 5A**) was similar to that of these chromosomes on the β-glucan content of wheat, determined with a Megazyme kit (**Figure 3A**). The addition of chromosome 5U reduced the ratio of DP3:DP4 glucan units released by lichenase from TOTAL-GOS. In addition, chromosomes 6U^g^ and 3U^b^ had decreasing effects on the DP3:DP4 ratio (**Figure 5B**), reflecting a higher ratio of larger polymers.

Differences in the structure of AX were determined after digestion with endoxylanase. AX molecules have a backbone of xylose residues which may be substituted with arabinose. They vary in structure, in terms of both the proportion and distribution of unsubstituted, monosubstituted, and disubstituted xylose residues. Digestion with a specific endoxylanase releases AXOS which can be separated and quantified by HPAEC, giving “fingerprints” for the samples. As the structures of the separated AXOS have been determined (Ordaz-Ortiz et al., 2005), the peak areas can also be used to compare the proportion of AXOS containing unsubstituted, monosubstituted, and disubstituted xylose residues. This comparison showed that TOTAL-AXOS was ∼50% lower in both *Ae. geniculata* and *Ae. biuncialis* than in wheat (**Figure 5C**). The proportion of substituted xylose residues was also significantly lower in both parental *Aegilops* accessions than in the wheat genotypes (**Figures 5D–F,H**). In addition, the ratio of monosubstituted to disubstituted xylose residues was almost twice as high in *Ae. geniculata* than in wheat or *Ae. biuncialis* (**Figure 5G**). The ratio of unsubstituted AXOS to total AXOS (**Figures 5C,D**) was increased by the addition of chromosomes 5M^g^, 1U^b^, 1U^b^6U^b^, 3M^b^, and 7M^b^, with monosubstituted AXOS being affected by chromosomes 2U^g^, 3-5U^g^, 2M, and 6M^g^ (**Figure 5E**) and disubstituted AXOS by chromosomes 4U^g^, 6M^g^, and 2M^b^ (**Figure 5F**). The ratio of monosubstituted to disubstituted AXOS was increased by the addition of chromosomes 2U^g^, 4-6U^g^, 2M, 3M^b^, and 7M^b^ (**Figure 5G**).

### Chromosomal Assignment of Genes Involved in the Biosynthesis of Storage Proteins, β-Glucan, or AX in *Aegilops*

In order to identify *Aegilops* homologs of the key genes responsible for the biosynthesis of storage proteins, β-glucan, or AX, a comparison was made between wheat or barley and the *Aegilops* genomes. As complete sequences of the U- and M-genomes of *Ae. geniculata* and *Ae. biuncialis* are not available, chromosome survey sequences of the *Ae. umbellulata* genomes were used for comparative analysis (see text footnote 3). A BLASTn search on the cDNA sequences of the key genes responsible for wheat grain storage proteins (**Table 1** and Supplementary Table 1) showed that most of the investigated genes (HMW glutenins, LMW glutenins, γ-gliadins) were assigned to the same homeologous group chromosomes (group 1) in the U-genome of *Ae. umbellulata* as in bread wheat (**Table 1** and Supplementary Table 1). The α-gliadin genes, which are assigned to group 6 chromosomes in bread wheat, were located on the 1U and 3U chromosomes of *Aegilops*. As for the genes involved in β-glucan biosynthesis (*OsCslF1-F2*, *HvCslF3-4*, *HvCslF6-10*, *HvCslF12-13*, *HvCslH1*), the *Aegilops* homologs were again assigned to the same homeologous group chromosomes (group 1, 2, 5, and 7) as in bread wheat (**Table 2** and Supplementary Table 2). This was also true for most of the genes responsible for AX biosynthesis, which were assigned to group 4 or 7 chromosomes (*TaGT43* family), group 3 chromosomes (*TaGT47* family), and group 2 chromosomes (*TaGT75* family) (**Table 2** and Supplementary Table 3). However, some *Aegilops* homologs were found on different chromosomes than the wheat genes. For example, gene *TaGT47-12* was assigned to the group 3 chromosomes of hexaploid wheat, while its *Aegilops* homolog was found on chromosome 6U. Differences in chromosomal assignments were also found for genes *HvCslF11*, *TaGT61-1*, *TaGT61-2*, and *TaGT75-4* (**Table 2** and Supplementary Tables 2, 3).

**Table 1:** Chromosomal assignment of genes responsible for grain storage protein biosynthesis in hexaploid wheat and *Ae. umbellulata*.

Function	Gene	Accession no.^∗^	Chromosome	
			
			*T. aestivum*	*Ae. umbellulata*
Protein biosynthesis				
HMW glutenins	Glu-1Ax1	X61009	1A	1U
	Glu-1Ax2	M22208.2	1A	1U
	Glu-B1-1b	X13927.3	1B	1U
	Glu-1D-1d	X12928.5	1D	1U
	Glu-D1-2b	X12929.2	1D	1U
		X03041.1	1D	1U
	Glu-1Ux	AF476961.1	–	1U
	Glu-1Uy	AF476962.1	–	1U
LMW glutenins		AB062868.1	1D	1U
		AB062872.1	1D	1U
		JX163862.1	1B	1U
		HM055909.1	1DS	1U
		Y17845.1	1BS	1U
		U86026.1	1DS	1U
		X13306.1	1DS	1U
		AB062875.1	1DS	1U
		U86028.1	1DS	1U
		X07747.1	1AS	1U
		AB062873.1	1DS	1U
γ-Gliadins	Group/pattern/subgroup			
	C10/C10-P1/SG-1	AJ937838.1	1DS	1U
	C9/C9-P2/SG-2	AF234646.1	1DS	1U
	C9/C9-P3/SG-3	FJ006638.1	1DS	1U
	C9/C9-P4/SG-4	FJ006605.1	1DS	1U
	C9/C9-P4/SG-6	AF234647.1	1BS	1U
	C9/C9-P4/SG-7	FJ006596.1	1DS	1U
	C8/C8-P5/SG-8	AF175312.1	1DS	1U
	C8/C8-P5/SG-9	AF120267.1	1DS	1U
	C8/C8-P5/SG-12	AF234649.1	1DS	1U
	C8/C8-P5/SG-13	AF234643.1	1AS	1U
	C7/C7-P6/SG-14	AJ416336.1	1DS	1U
	C7/C7-P7/SG-15	M16064.1	1DS	1U
α-Gliadins		AJ133612.1	6AS	1U
		DQ166377.1	6AS	1U
		K03074.1	2BS	1U
		M11075.1	6AS	1U, 2U
		U08287.1	6AS	1U, 3U
		X01130.1	6AS	1U, 3U
		U50984.1	6AS	1U, 3U
		X02539.1	6AS	1U, 3U


*^∗^NCBI (https://www.ncbi.nlm.nih.gov/)*

**Table 2:** Chromosomal assignment of genes responsible for β-glucan and arabinoxylan biosynthesis in hexaploid wheat and *Ae. umbellulata*.

Function	Gene	Accession no.^∗^	Chromosome	
			
			*T. aestivum*	*Ae. umbellulata*
β-Glucan biosynthesis	*OsCslF1*	AF432502.1	2AS, 2BS	2U
	*OsCslF2*	AF432503.1	2BL	2U
	*HvCslF3*	EU267179.1	2AS, 2BS, 2DS	2U
	*HvCslF4*	EU267180.1	2AS, 2BS	2U
	*HvCslF6*	EU267181.1	7DL	7UL
	*HvCslF7*	EU267182.1	5BL	5U
	*HVCslF8*	EU267183.1	2AS, 2BS, 2DS	2U
	*HVCslF9*	EU267184.1	1AS, 1BS, 1DS	1U
	*HvCslF10*	EU267185.1	2AS, 2BS, 2DS	2U
	*HvCslF11*	–	7DL, 7BL	6U
	*HvCslF12*	–	2AS, 2BS, 2DS	2U
	*HvCslF13*	–	2AL, 2BL	2U
	*HvCslH1*	–	2AL, 2BL, 2AL	2U
Arabinoxylan biosynthesis	*TaGT43-2D*	HF913567.1	4AS	4U
	*TaGT43-2B*	HF913568.1	4AS	4U
	*TaGT43-2A*	HF913569.1	4AS	4U
	*TaGT43-4*	HM236487.1	7AL, 7BL, 7DL	7UL
	*TaGT47-2B*	HF913570.1	3B	3U
	*TaGT47-2D*	HF913571.1	3AL	3U
	*TaGT47-2A*	HF913572.1	3AL	3U
	*TaGT47-12*	HM236486.1	3AL, 3B, 3DL	6U
	*TaGT47-13*	HM236485.1	3AL, 3B, 3DL	3U
	*TaGT61-1*	FR873610.1	1BL	6U
	*TaGT61-2*	FR846232.1	6AL	4U
	*TaGT75-1*	HM236488.1	2AL, 2BL, 2DL	2U
	*TaGT75-4*	HM236489.1	4AL, 4BS, 4DS	6U
	*TaBAHD1A*	Traes_3AS_75E04A7F4^∗∗^	3AS	3U


*^∗^NCBI (https://www.ncbi.nlm.nih.gov/); ^∗∗^EnsemblPlants (http://plants.ensembl.org/)*

## Discussion

Despite their high nutritional value, very few studies have examined the potential of wild genetic resources to improve the content and composition of the edible fiber components in wheat grain (Marcotuli et al., 2015, 2016). *Ae. biuncialis* and *Ae. geniculata* exhibited substantial genetic diversity in their protein and fiber fractions relative to the wheat parents used in this study, which indicates that the effect of the chromosome additions on the quality of these components could be reliably studied. Therefore, the present study focuses on the ability of the U- and M-genome chromosomes of *Aegilops* to modify the amount and composition of storage proteins, AX and β-glucan in bread wheat. Furthermore, this work also provides information on the chromosomal assignment of potential wild alleles of key genes responsible for the biosynthesis of proteins and DFs.

### Proteins

The addition of the group 1 chromosomes of *Ae. geniculata* (1U^g^ and 1M^g^) to bread wheat was found to increase the proportion of insoluble glutenins relative to total glutenins (%UPP). These chromosomes also increased the ratio of glutenins to gliadins (Glu/Gli) (**Figures 2C,D**). These results are in line with the observations of Garg et al. (2016), who used the same set of Chinese Spring–*Ae. geniculata* addition lines and found that chromosome 1M^g^ exhibited greater dough strength than the parental wheat. Wheat gluten protein has been studied in great detail for more than a half century, with the first genetic studies dating back to the 1960s. It has been established that three major groups of wheat gluten proteins (LMW subunits of glutenin, ω-gliadins, and γ-gliadins) are encoded by genes on the short arms of the group 1 chromosomes of all three genomes of wheat (A, B, and D), while the HMW subunits of glutenin are encoded by genes on the long arms of the same chromosomes. A further group of gluten proteins, the α-gliadins, are encoded by genes on the short arms of the group 6 chromosome (Payne, 1987; Shewry et al., 2003a,b, 2009).

Wild homologs of the gluten protein genes were detected on the 1U chromosome sequence contigs of *Ae. umbellulata* (**Table 1** and Supplementary Table 1), consistently with previous studies on *Ae. geniculata* (Medouri et al., 2015). A recent comparative analysis of individual flow-sorted chromosomes of *Ae. umbellulata*, *Ae. comosa*, and wheat indicated that chromosomes 1U and 1M are syntenic with the group 1 chromosomes of hexaploid wheat at the macro level (Molnár et al., 2016), suggesting that the 1M^g^ chromosome of *Ae. geniculata* may also contain wild alleles of gluten protein genes.

### β-Glucan

The proportion of dietary fiber components in both *Aegilops* species was more similar to that in oats, barley, and *Brachypodium* than to that in wheat, with higher proportions of β-glucan than of AX. There is considerable interest in increasing the content of β-glucan in wheat flour due to its known health benefits (Buttriss and Stokes, 2008; Anderson et al., 2009; Tighe et al., 2010). The addition of chromosome 5U^g^ or group 7 chromosomes from the U and M genomes of *Ae. geniculata* and *Ae. biuncialis* were able to significantly increase the β-glucan content of wheat (**Figure 3A**) across different growth conditions. The addition of the 1U^g^, 7U^g^ (or 7M^g^), and 3M^g^ chromosomes also increased TOTAL-GOS (**Figure 5A**). Chromosome 5U was also found to reduce the quantitative ratio of DP3:DP4 glucan units (**Figure 5B**). Earlier studies indicated that the addition of 0–5% β-glucans to bread flour significantly reduced dough extensibility and loaf volume (Brennan and Cleary, 2007), while the solubility of the fibers was decreased by higher β-glucan levels (Izydorczyk and Dexter, 2008), but increased by a lower DP3:DP4 ratio (Izydorczyk and Dexter, 2008). The two opposite effects probably result in the solubility of β-glucan remaining nearly constant in the *Aegilops* addition lines, while little changes could be expected in their processing properties.

Cellulose synthase-like (*Csl*) genes are candidates to encode enzymes that synthetize the backbone of various non-cellulosic cell wall polysaccharides (Doblin et al., 2009). They have been classified into nine gene families, designated *CslA* to *CslJ*, of which the *CslF*, *CslH*, and *CslJ* families are restricted to cereals, although the *CslJ* group is not found in rice (*Oryza sativa* L.) or *Brachypodium distachyon* L. (Doblin et al., 2010). Expression in transgenic *Arabidopsis thaliana* L. plants revealed that the barley *CslF* and *CslH* families are probably involved in β-glucan synthesis (Burton et al., 2006; Doblin et al., 2009). Comparative genomic studies have shown that barley has 10 *CslF* family members; *HvCslF3*, *HvCslF4*, *HvCslF8*, *HvCslF10*, *HvCslF12*, and *HvCslF13* clustered on chromosome 2H, *HvCslF9*, located on 1H, *HvCslF7*, located on 5H and *HvCslF6* and *HvCslF11*, located on 7H (Burton et al., 2008; Schreiber et al., 2014). Among the *HvCslF* genes of barley, *HvCslF6* and *HvCslF9* have the highest levels of mRNA transcripts in developing barley endosperms (Burton et al., 2008) and map to loci near the centromeres of chromosomes 7H and 1H, respectively, which are close to quantitative trait loci (QTL) for the β-glucan content of barley grain (Igartua et al., 2002; Molina-Cano et al., 2007; Burton et al., 2008). Taketa et al. (2012) analyzed barley mutants lacking β-glucan and showed that the *HvCslF6* gene had a unique role and was the key determinant controlling the biosynthesis of β-glucan, while Nemeth et al. (2010) demonstrated the role of the *CslF6* gene in wheat β-glucan synthesis by RNAi suppression in grain of transgenic plants.

The β-glucan content of wheat–*Aegilops* chromosome addition lines was consistent with the locations of putative β-glucan synthase genes in the U genome of *Ae. umbellulata*. A sequence similarity search on the barley *Cellulose-synthase-like F* (*CslF*) gene family showed that the *Aegilops* orthologs of the *CslF* family members were located on the same homeologous group chromosomes as in barley. More precisely, homologs of the *CslF6* genes of barley were present on chromosome 7U and those of *CslF9* on chromosome 1U, while homologs of other *CslF* genes were located on chromosomes 2U and 5U (**Table 2** and Supplementary Table 2). Previous results (Cseh et al., 2011, 2013) indicated that the chromosome 7H-mediated transfer of *HvCslF6* gene from barley cv. Manas to wheat significantly increased the level of β-glucan in the grains. While the increase was statistically significant, it should be noted that the β-glucan level content of the 7H addition line was moderate compared to that of the parental barley genotype (Cseh et al., 2011). An explanation for the moderate increase in the β-glucan level of wheat–barley 7H addition line could be that the barley QTL represented on chromosome 7H is only a part of the genomic regions needed for a more efficient synthesis of β-glucan (Houston et al., 2014; Shu and Rasmussen, 2014) and it is not sufficient to drive higher β-glucan levels. This may also apply to the wheat–*Aegilops* addition lines 5U^g^ or 7U^g^, 7M^g^ and 7M^b^ (and for 1U^b^ under field conditions) where the increase in the β-glucan amount was also moderate.

A homolog of *HvCslF6* gene was also identified on chromosome 7U, which further supports the fact that the addition of group 7 chromosomes from *Ae. geniculata* and *Ae. biuncialis* to wheat increases the grain β-glucan level. A putative ortholog of the HvCslF7 gene was detected on chromosome 5U, with an increased β-glucan level in the wheat–*Ae. geniculata* 5U^g^ addition line. These results suggest that the variant of *HvCslF7* may have a role in grain β-glucan synthesis of *Aegilops* (Burton et al., 2008).

However, further genome-wide association studies on a diverse population of *Ae. geniculata*, or *Ae. biuncialis* would help to identify the chromosomal locations of QTL responsible for high β-glucan levels in grain endosperm. The addition lines contain a whole homeologous chromosome pair from *Aegilops* and, beside the desirable genomic regions, they also contain many other genes which may affect grain development and composition. The elimination of excess alien chromatin by the production of wheat–*Aegilops* translocation lines containing desirable QTL and their pyramiding into one genotype could be used to increase β-glucan content.

### Arabinoxylan

The observation that the addition of chromosomes 5U^g^, 7U^g^ and 1U^b^ to wheat increases the total pentosan content, while the addition of chromosomes 3U^g^, 4U^g^, 5U^g^, 6U^g^, 7U^g^, 5M^g^, 7M^g^, 2M^b^ and 7M^b^ increases the water-soluble pentosan fraction (**Figures 3B,C**) indicates that goatgrasses are a promising source of genes to increase the AX content of wheat. As well as their health benefits, both water-soluble and unsoluble AX result in higher water absorption, dough development time and loaf volume (Biliaderis et al., 1995; Courtin and Delcour, 2002). Soluble AX contributes to gas bubble formation during baking while insoluble AX destabilizes it (Courtin and Delcour, 2002).

Many efforts have been made to identify genes controlling the biosynthesis of AXs in wheat and several mapping populations have been used to identify QTL for AX in wheat. A major QTL was identified on chromosome 1B (Martinant et al., 1999), which explained 59% of the phenotypic variation in WE-AX content and viscosity (Charmet et al., 2009). Quraishi et al. (2011) identified seven loci (chromosomes 1B, 3A, 3D, 5B, 6B, 7A, and 7B) by association genetics, of which three (chromosomes 1B, 3D, and 6B) corresponded to consensus meta-QTL based on data from seven crosses. These authors also identified candidate genes for the future improvement of grain fiber. Using bioinformatic approaches, Mitchell et al. (2007) identified genes for AX synthesis in glycosyltransferase (GT) families 43, 47, and 61, while Zeng et al. (2010) identified GT75 with a combination of proteomics and transcriptomics analyses. In wheat, most GT genes occur in multiple forms, with three homeoalleles of each form being present on the A, B, and D genomes (Mitchell et al., 2007; Wan et al., 2008; Pellny et al., 2012). RNAi suppression of the expression of these homeoalleles demonstrated that GT43 and GT47 encode subunits of β-1,4-xylan synthase and that GT61 encodes a α-(1,3)-arabinosyltransferase (Anders et al., 2012; Lovegrove et al., 2013). Suppression of either TaGT43_2 or TaGT47 resulted in a 40–50% decrease in total AX but increased Araf residues substitution, with a 50% decrease in cell-wall thickness (Lovegrove et al., 2013). Similarly, the suppression of GT61 (renamed TaXAT1) resulted in a 70–80% decrease in the amount of α-(1,3) linked Araf in the AX of mature starchy endosperm (Anders et al., 2012). Decreases in extract viscosity were observed in all transgenic lines, with greater effects in the TaGT43_2 and TaGT47_2 RNAi lines (located on chromosomes 4ABD and 3ABD of wheat, respectively) than in the TaXAT1 RNAi lines (Freeman et al., 2016). These effects were explained by decreases in the amount and chain length of WE-AX.

Putative orthologs of wheat *GT43*, *GT47* and *GT61* and *GT75* genes were identified on chromosomes 3U (*TaGT47-2B*, *TaGT47-2D*, *TaGT47-2A*, *TaGT47-13*), 4U (*TaGT43-2B*, *TaGT43-2A*, *TaGT61-2*), 6U (*TaGT47-12*, *TaGT61-1*, *TaGT75-4*), and 7U (*TaGT43-4*) in the U genome of *Ae. umbellulata*, which is consistent with the present observation that the addition of chromosome 7U^g^ increased total pentosan, while the addition of chromosomes 3U^g^, 4U^g^, 6U^g^, and 7U^g^ increased WE-pentosan (**Figures 3B,C**, **Table 2**, and Supplementary Table 3). It should be noted that the addition of *Ae. geniculata* chromosome 5U^g^ increased the total- and WE-pentosan content in wheat even if no orthologs of wheat GT genes were identified on chromosome 5U. However, the increased AX content of the 5U^g^ addition line is consistent with a recent study by Marcotuli et al. (2015), who used GWAS to identify three QTL strongly associated with AX content on chromosome 5A of tetraploid wheat. The possible reason for this discrepancy could be that the chromosome survey sequences do not cover the whole of the *Ae. umbellulata* genome (data not shown).

The ratio of AXOS released by endoxylanase digestion was affected by *Ae. biuncialis* chromosomes 2M^b^, 3M^b^, and 7M^b^ in wheat, as their addition increased the ratio of monosubstituted to disubstituted AXOS. However, this increase in the M/D ratio was due to an increase in the amount of monosubstituted AXOS in addition line 2M^b^, but due to a decrease in the amount of disubstituted AXOS in the addition lines 3M^b^ and 7M^b^. These results indicate that AX biosynthesis could be modified by these *Aegilops* chromosomes. However, other genes were also observed to have smaller effects (**Figure 5G**). Interestingly, several enzymes involved in the AX biosynthesis pathway were identified on the group 2, 3, and 7 chromosomes of wheat (Group 2: glucuronosyltransferase, *cis*-zeatin *O*-glucosyltransferase 1, glycosyltransferase; group 3: glycosyl hydrolase; group 7: 1,3-β-D-glucan synthase, Glycosyl hydrolase, β-1,4-endoglucanase) (Marcotuli et al., 2015). The fact that the *Ae. geniculata* and *Ae. biuncialis* accessions exhibited significant phenotypic variations for the grain β-glucan and AX content supports the notion that these species have considerable genetic variability for improving the edible fiber content of bread wheat.

## Conclusion

In conclusion, the present study highlighted the ability of *Aegilops* chromosomes 5U^g^, 7U^g^, 7M^g^, and 7M^b^ to increase the β-glucan and 5U^g^, 5M^g^, and 7M^b^ to improve WE-AX content of hexaploid wheat grains. After the selection of suitable *Aegilops* crossing partners, these *Aegilops* chromosomes could be promising candidates for chromosome-mediated gene transfer and chromosome engineering programs aimed to improve the DF content of wheat. Furthermore, the chromosomal assignment of *Aegilops* orthologs for genes influencing grain β-glucan and AX content provides a foundation for further genome-wide association studies to identify QTL responsible for the amount and composition of edible fiber in these *Aegilops* species. The study will thus contribute to the more efficient use of wild wheat relatives in alien introgression breeding programs to obtain wheat varieties with increased fiber content, especially β-glucan and improved health benefits, in general.

## Author Contributions

Plant materials were developed by MM-L, IM, ÉD, and AF. Cytogenetic checking was carried out by AF. Experimentation and data evaluation were carried out by MR, AL, and PS. Background for research was established by IM, PS, LL, and ZB. Sequence data of the U genome of *Aegilops umbellulata* were made available by JD. Blast searches were carried out by IM. Paper was written by MR, IM, AL, ÉD, and PS.

## Conflict of Interest Statement

The authors declare that the research was conducted in the absence of any commercial or financial relationships that could be construed as a potential conflict of interest.

## Footnotes

1 www.jiffygroup.com

2 http://olomouc.ueb.cas.cz/projects/Aegilops_umbellulata

3 https://www.ncbi.nlm.nih.gov

4 http://plants.ensembl.org/

## Supplementary Material

**FIGURE 1 |** Degradation products obtained after xylanase and lichenase treatment of flour samples and run on HPAEC-PAD [*y*-axis: PAD response (μV); *x*-axis: Time (min)].

**FIGURE 2 |** Thousand kernel weight and compositional properties of mature grains of two lines of bread wheat (cv. Chinese Spring and Mv9kr1 line), two *Aegilops* species (*Ae. geniculata* and *Ae. biuncialis*), and wheat–*Aegilops* chromosome addition lines grown on the field in 2012/2013 season. **(a)** thousand kernel weight, **(b)** protein, **(c)** β-glucan content, **(d)** TOT-pentosan, **(e)** WE-pentosan content. LSD, least significant difference; TOT, total; WE, water-extractable. ^∗^Significantly higher than the wheat (*T. aestivum*) control.

## Abbreviations

Araf: α-L-arabinofuranosyl
A/X: ratio of arabinose to xylose
AXOS: arabinoxylan oligosaccharides
D: (XA^2+3^XX) + (XA^3^A^2+3^X) + (XA^3^XA^2+3^XX) sum of disubstituted AXOS
DP: degree of polymerization
GOS: glucooligosaccharides
HPAEC: high-performance anion exchange chromatography
M: (XA^3^XX) + 2(XA^3^A^3^XX) + 2(XA^3^XA^3^XX) + (XA^3^A^2+3^X) + (XA^3^XA^2+3^XX) sum of monosubstituted AXOS
PAD: pulsed amperometric detection
TKW: thousand kernel weight
TOT: X + XX + XXX + XA^3^XX + XA^3^A^3^XX + XA^3^XA^3^XX + (XA^2+3^XX) + (XA^3^A^2+3^XX) + (XA^3^XA^2+3^XX) sum of all AXOS
TOT-AX: total arabinoxylan
US: X + XX + XXX sum of unsubstituted AXOS
WE-AX: water extractable arabinoxylan

## References

[B1] AACC International (1995). *Approved Methods 32-23.01. Beta-Glucan Content of Barley and Oats – Rapid Enzymatic Procedure.* St. Paul, MN: AACC International.

[B2] Ahmadpoor F. Asghari-Zakaria R. Firoozi B. Shahbazi H. (2014). Investigation of diversity in *Aegilops biuncialis* and *Aegilops umbellulata* by A-PAGE. *Nat. Prod. Res.* 28 1626–1636. 10.1080/14786419.2014.931392 25066882

[B3] Anders N. Wilkinson M. D. Lovegrove A. Freeman J. Tryfona T. Pellny T. K. (2012). Glycosyl transferases in family 61 mediate arabinofuranosyl transfer onto xylan in grasses. *Proc. Natl. Acad. Sci. U.S.A.* 109 989–993. 10.1073/pnas.1115858109 22215597 PMC3271882

[B4] Anderson J. W. Baird P. Davis R. H. Jr. Ferreri S. Knudtson M. Koraym A. (2009). Health benefits of dietary fiber. *Nutr. Rev.* 67 188–205. 10.1111/j.1753-4887.2009.00189.x 19335713

[B5] Andersson A. A. M. Kamal-Eldin A. Fras A. Boros D. Aman P. (2008). Alkylresorcinols in wheat varieties in the HEALTHGRAIN diversity screen. *J. Agric. Food Chem.* 56 9722–9725. 10.1021/jf8011344 18921971

[B6] Bálint A. Kovács G. Erdei L. Sutka J. (2001). Comparison of the Cu, Zn, Fe, Ca and Mg contents of the grains of wild, ancient and cultivated wheat species. *Cereal Res. Commun.* 29 375–382.

[B7] Bamforth C. W. (2010). The enzymology of cell wall breakdown during malting and mashing: An Overview. *Tech. Q. Mast. Brew. Assoc. Am.* 47 309–321. 10.1094/TQ-47-1-0309-01

[B8] Bandou H. Rodriguez-Quijano M. Carrillo J. M. Branlard G. Zaharieva M. Monneveux P. (2009). Morphological and genetic variation in *Aegilops geniculata* from Algeria. *Plant Syst. Evol.* 277 85–97. 10.1007/s00606-008-0106-z

[B9] Batey I. L. Gupta R. B. MacRitchie F. (1991). Use of size-exclusion high performance liquid chromatography in the study of wheat flour proteins: an improved chromatographic procedure. *Cereal Chem.* 68 207–209.

[B10] Bedford M. R. Schulze H. (1998). Exogenous enzymes for pigs and poultry. *Nutr. Res. Rev.* 11 91–114. 10.1079/NRR19980007 19087461

[B11] Biliaderis C. G. Izydorczyk M. S. Rattan O. (1995). Effect of arabinoxylans on bread-making quality of wheat flours. *Food Chem.* 53 165–171. 10.1016/0308-8146(95)90783-4

[B12] Bonnand-Ducasse M. Della Valle G. Lefebvre J. Saulnier L. (2010). Effect of wheat dietary fibres on bread dough development and rheological properties. *J. Cereal Sci.* 52 200–206. 10.1016/j.jcs.2010.05.006

[B13] Boros D. Lukaszewski A. J. Aniol A. Ochodzki P. (2002). Chromosome location of genes controlling the content of dietary fibre and arabinoxylans in rye. *Euphytica* 128 1–8. 10.1023/A:1020639601959

[B14] Brennan C. S. Cleary L. J. (2007). Utilisation Glucagel^®^in the β-glucan enrichment of breads A physicochemical and nutritional evaluation. *Food Res. Int.* 40 291–296. 10.1016/j.foodres.2006.09.014

[B15] Brouns F. J. P. H. van Buul V. J. Shewry P. R. (2013). Does wheat make us fat and sick? *J. Cereal Sci.* 58 209–215. 10.1016/j.jcs.2013.06.002

[B16] Buksa K. Nowotna A. Ziobro R. (2016). Application of cross-linked and hydrolyzed arabinoxylans in baking of model rye bread. *Food Chem.* 192 991–996. 10.1016/j.foodchem.2015.07.104 26304439

[B17] Burton R. A. Jobling S. A. Harvey A. J. Shirley N. J. Mather D. E. Bacic A. (2008). The genetics and transcriptional profiles of the cellulose synthase-like HvCslF gene family in barley. *Plant Physiol.* 146 1821–1833. 10.1104/pp.107.114694 18258691 PMC2287353

[B18] Burton R. A. Wilson S. M. Hrmova M. Harvey A. J. Shirley N. J. Medhurst A. (2006). Cellulose synthase-like CslF genes mediate the synthesis of cell wall (1,3;1,4)-beta-D-glucans. *Science* 311 1940–1942. 10.1126/science.1122975 16574868

[B19] Buttriss J. L. Stokes C. S. (2008). Dietary fibre and health: an overview. *Nutr. Bull.* 33 186–200. 10.1111/j.1467-3010.2008.00705.x

[B20] Cavallero A. Empilli S. Brighenti F. Stanca A. M. (2002). High (1-3, 1-4)-β-glucan fractions in bread making and their effect on human glycemic response. *J. Cereal Sci.* 36 59–66. 10.1006/jcrs.2002.0454

[B21] Charmet G. Masood-Quraishi U. Ravel C. Romeuf I. Rakszegi M. Guillon F. (2009). Genetics of dietary fibre in bread wheat. *Euphytica* 170 155–168. 10.1007/s10681-009-0019-0

[B22] Cleary L. J. Andersson R. Brennan C. S. (2007). The behaviour and susceptibility to degradation of high and low molecular weight barley β-glucan in wheat bread during baking and in vitro digestion. *Food Chem.* 102 889–897. 10.1016/j.foodchem.2006.06.027

[B23] Colmer T. D. Flowers T. J. Munns R. (2006). Use of wild relatives to improve salt tolerance in wheat. *J. Exp. Bot.* 57 1059–1078. 10.1093/jxb/erj124 16513812

[B24] Courtin C. M. Delcour J. (1998). Physicochemical and bread-making properties of low molecular weight wheat-derived Arabinoxylans. *J. Agric. Food Chem.* 46 4066–4073. 10.1021/jf980339t

[B25] Courtin C. M. Delcour J. (2002). Arabinoxylans and endoxylanases in wheat flour bread-making. *J. Cereal Sci.* 35 225–243. 10.1006/jcrs.2001.0433

[B26] Cseh A. Kruppa K. Molnár I. Rakszegi M. Doležel J. Molnár-Láng M. (2011). Characterization of a new 4BS.7HL wheat/barley translocation line using GISH, FISH and SSR markers and its effect on the β-glucan content of wheat. *Genome* 54 795–804. 10.1139/g11-044 21919737

[B27] Cseh A. Soós V. Rakszegi M. Türkösi E. Balázs E. Molnár-Láng M. (2013). Expression of HvCslF9 and HvCslF6 barley genes in the genetic background of wheat and their influence on the wheat β-glucan content. *Ann. Appl. Biol.* 163 142–150. 10.1111/aab.12043

[B28] Cui W. Wood P. J. (2000). “Relationships between structural features, molecular weight and rheological properties of cereal beta-D- glucans,” in *Hydrocolloids, PT 1: Physical Chemistry and Industrial Application of Gels, Polysaccharides and Proteins*, ed. Nishinari K. (Amsterdam: Elsevier), 159–168.

[B29] Cui W. Wood P. J. Blackwell B. Nikiforuk J. (2000). Physicochemical properties and structural characterization by two-dimensional NMR spectroscopy of wheat β-D-glucan—comparison with other cereal β-D-glucans. *Carbohydr. Polym.* 41 249–258. 10.1016/S0144-8617(99)00143-5

[B30] Cyran M. Rakowska M. Miazga D. (1996). Chromosomal location of factors affecting content and composition of nonstarch polysaccharides in wheat-rye addition lines. *Euphytica* 89 153–157. 10.1007/BF00015732

[B31] Dai S. F. Zhao L. Xue X. F. Jia Y. N. Liu D. C. Pu Z. J. (2015). Analysis of high-molecular-weight glutenin subunits in five amphidiploids and their parental diploid species *Aegilops umbellulata* and *Aegilops uniaristata*. *Plant Genet. Resour.* 13 186–189. 10.1017/S1479262114000719

[B32] Doblin M. S. Pettolino F. Bacic A. (2010). Plant cell walls: the skeleton of the plant world. *Funct. Plant Biol.* 37 357–381. 10.1071/FP09279

[B33] Doblin M. S. Pettolino F. A. Wilson S. M. Campbell R. Burton R. A. Fincher G. B. (2009). A barley cellulose synthase-like CSLH gene mediates (1,3;1,4)-beta-D-glucan synthesis in transgenic Arabidopsis. *Proc. Natl. Acad. Sci. U.S.A.* 106 5996–6001. 10.1073/pnas.0902019106 19321749 PMC2667043

[B34] Doležel J. Vrána J. Cápal P. Kubaláková M. Burešová V. Šimková H. (2014). Advances in plant chromosome genomics. *Biotechnol. Adv.* 32 122–136. 10.1016/j.biotechadv.2013.12.011 24406816

[B35] Douglas S. G. (1981). A rapid method for the determination of pentosans in wheat flour. *Food Chem.* 7 139–145. 10.1016/0308-8146(81)90059-5

[B36] Dulai S. Molnár I. Szopkó D. Darkó É Vojtkó A. Sass-Gyarmati A. (2014). Wheat-*Aegilops biuncialis* amphiploids have efficient photosynthesis and biomass production during osmotic stress. *J. Plant Physiol.* 171 509–517. 10.1016/j.jplph.2013.11.015 24655386

[B37] Farkas A. Molnár I. Dulai S. Rapi S. Oldal V. Cseh A. (2014). Increased micronutrient content (Zn, Mn) in the 3Mb(4B) wheat-*Aegilops biuncialis* substitution and 3Mb.4BS translocation identified by GISH and FISH. *Genome* 57 61–67. 10.1139/gen-2013-0204 24702063

[B38] Finnie S. M. Bettge A. D. Morris C. F. (2006). Influence of cultivar and environment on water-soluble and water-insoluble arabinoxylans in soft wheat. *Cereal Chem.* 83 617–623. 10.1094/CC-83-0617

[B39] Frederix S. A. Van Hoeymissen K. Courtin C. M. Delcour J. A. (2004). Water-extractable and water-unextractable arabinoxylans affect gluten agglomeration behavior during wheat flour gluten-starch separation. *J. Agric. Food Chem.* 52 7950–7956. 10.1021/jf049041v 15612781

[B40] Freeman J. Lovegrove A. Wilkinson M. D. Saulnier L. Shewry P. R. Mitchell R. A. C. (2016). Effect of suppression of arabinoxylan synthetic genes in wheat endosperm on chain length of arabinoxylan and extract viscosity. *Plant Biotechnol. J.* 14 109–116. 10.1111/pbi.12361 25819752 PMC5098169

[B41] Friebe B. Jiang J. Raupp W. J. McIntosh R. A. Gill B. S. (1996). Characterization of wheat alien translocations conferring resistance to diseases and pests: current status. *Euphytica* 71 59–87. 10.1007/BF00035277

[B42] Friebe B. Tuleen N. A. Gill B. S. (1999). Development and identification of a complete set of *Triticum aestivum*–Ae. geniculata chromosome addition lines. *Genome* 42 374–380. 10.1139/gen-42-3-374

[B43] Garg M. Tsujimoto H. Gupta R. K. Kumar A. Kaur N. Kumar R. (2016). Chromosome specific substitution lines of *Aegilops geniculata* alter parameters of bread making quality of wheat. *PLoS ONE* 11:e0162350 10.1371/journal.pone.0162350 PMC5068752 27755540

[B44] Gebruers K. Dornez E. Bedo Z. Rakszegi M. Fras A. Boros D. (2010). Environment and genotype effects on the content of dietary fiber and its components in wheat in the HEALTHGRAIN diversity screen. *J. Agric. Food Chem.* 58 9353–9361. 10.1021/jf100447g 20462191

[B45] Gebruers K. Dornez E. Boros D. Fras A. Dynkowska W. Bedo Z. (2008). Variation in the content of dietary fiber and components thereof in wheats in the HEALTHGRAIN diversity screen. *J. Agric. Food Chem.* 56 9740–9749. 10.1021/jf800975w 18921978

[B46] Heinio R. L. Noort M. W. J. Katina K. Alam S. A. Sozer N. de Kock H. L. (2016). Sensory characteristics of wholegrain and bran-rich cereal foods - A review. *Trends Food Sci. Technol.* 47 25–38. 10.1016/j.tifs.2015.11.002

[B47] Hesselman K. Elwinger K. Nilsson M. Thomke S. (1981). The effect of beta-glucanase supplementation, stage of ripeness, and storage treatment of barley in diets fed to broiler chickens. *Poult. Sci.* 60 2664–2671. 10.3382/ps.0602664

[B48] Hoffmann R. A. Leeflang B. R. de Barse M. M. J. Kamerling J. P. Vliegenthart J. F. G. (1991). Characterisation of 1H-N.M.R. spectroscopy of oligosaccharides, derived from arabinoxylans of white endosperm of wheat, that contain the elements -4) [α-L-Araf-(1-3)]-β-D-Xylp-(1-or-4)[α-L-Araf-(1-2)][α-L-Araf-(1-3)]-β-D-Xylp-(1-. *Carbohydr. Res.* 221 63–81. 10.1016/0008-6215(91)80049-S 1816926

[B49] Houston K. Russell J. Schreiber M. Halpin C. Oakey H. Washington J. M. (2014). A genome wide association scan for (1,3;1,4)-β-glucan content in the grain of contemporary 2-row spring and winter barleys. *BMC Genomics* 15:907 10.1186/1471-2164-15-907 PMC4213503 25326272

[B50] Igartua E. Hayes P. M. Thomas W. T. B. Meyer R. Mather D. E. (2002). Genetic control of quantitative grain and malt quality traits in barley. *J. Crop Prod.* 5 131–164. 10.1300/J144v05n01_06

[B51] International Association for Cereal Science and Technology ICC 105/2 (1995). *Determination of Crude Protein in Cereals and Cereal Products for Food and for Feed.* Vienna: International Association for Cereal Science and Technology.

[B52] International Association for Cereal Science and Technology ICC 166 (1998). *Determination of β-glucan in Barley, Oat and Rye.* Vienna: International Association for Cereal Science and Technology.

[B53] Izydorczyk M. S. Biliaderis C. G. (1994). Studies on the structure of wheat-endosperm arabinoxylans. *Carbohydr. Polym.* 24 61–71. 10.1016/0144-8617(94)90118-X

[B54] Izydorczyk M. S. Dexter J. E. (2008). Barley β-glucans and arabinoxylans: Molecular structure, physicochemical properties, and uses in food products – a Review. *Food Res. Int.* 41 850–868. 10.1016/j.foodres.2008.04.001

[B55] Jiang G. Vasanthan T. (2000). MALDI-MS and HPLC quantification of oligosaccharides of lichenase-hydrolyzed water-soluble b-glucan from ten barley varieties. *J. Agric. Food Chem.* 48 3305–3310. 10.1021/jf0001278 10956106

[B56] Jones J. M. Adams J. Harriman C. Miller C. Van der Kamp J. W. (2015). Nutritional impacts of different whole grain milling techniques: a review of milling practices and existing data. *Cereal Foods World* 60 130–139. 10.1094/CFW-60-3-0130

[B57] Kozub N. A. Sozinov I. A. Xynias I. N. Sozinov A. A. (2011). Allelic variation at high-molecular-weight glutenin subunit loci in *Aegilops biuncialis* Vis. *Russ. J. Genet.* 47 1078–1083. 10.1134/S1022795411090092 22117406

[B58] Lafiandra D. Riccardi G. Shewry P. R. (2014). Improving cereal grain carbohydrates for diet and health. *J. Cereal Sci.* 59 312–326. 10.1016/j.jcs.2014.01.001 24966450 PMC4064937

[B59] Larroque O. R. Békés F. (2000). Rapid size-exclusion chromatography analysis of molecular size distribution for wheat endosperm protein. *Cereal Chem.* 77 451–453. 10.1094/CCHEM.2000.77.4.451

[B60] Lazaridou A. Biliaderis C. G. (2007). Molecular aspects of cereal β-glucan functionality: physical properties, technological applications and physiological effects. *J. Cereal Sci.* 46 101–118. 10.1016/j.jcs.2007.05.003

[B61] Lazaridou A. Biliaderis C. G. Izydorczyk M. S. (2003). Molecular size effects on rheological properties of oat beta-glucans in solution and gels. *Food Hydrocoll.* 17 693–712. 10.1016/S0268-005X(03)00036-5

[B62] Lazaridou A. Biliaderis C. G. Micha-Screttas M. Steele B. R. (2004). A comparative study on structure-function relations of mixed linkage (1-3), (1-4) linear β-D-glucans. *Food Hydrocoll.* 18 837–855. 10.1016/j.foodhyd.2004.01.002

[B63] Li S. Morris C. F. Bettge A. D. (2009). Genotype and environment variation for arabinoxylans in hard winter and spring wheats of the US Pacific Northwest. *Cereal Chem.* 86 88–95. 10.1094/CCHEM-86-1-0088

[B64] Lovegrove A. Wilkinson M. D. Freeman J. Pellny T. K. Tosi P. Saulnier L. (2013). RNA interference suppression of genes in glycosyl transferase families 43 and 47 in wheat starchy endosperm causes large decreases in arabinoxylan content. *Plant Physiol.* 163 95–107. 10.1104/pp.113.222653 23878080 PMC3762668

[B65] Marcotuli I. Houston K. Schwerdt J. G. Waugh R. Fincher G. B. Burton R. A. (2016). Genetic diversity and genome wide association study of β-glucan content in tetraploid wheat grains. *PLoS ONE* 11:e0152590 10.1371/journal.pone.0152590 PMC4821454 27045166

[B66] Marcotuli I. Houston K. Waugh R. Fincher G. B. Burton R. A. Blanco A. (2015). Genome wide association mapping for arabinoxylan content in a collection of tetraploid wheats. *PLoS ONE* 10:e0132787 10.1371/journal.pone.0132787 PMC4503733 26176552

[B67] Mares D. J. Stone B. A. (1973). Studies on wheat endosperm. I. Chemical composition and ultrastructure of the cell walls. *Aust. J. Biol. Sci.* 26 793–812. 10.1071/BI9730793

[B68] Margulies M. Egholm M. Altman W. E. Attiya S. Bader J. S. Bemben L. A. (2005). Genome sequencing in microfabricated high-density picolitre reactors. *Nature* 437 376–380. 10.1038/nature03959 16056220 PMC1464427

[B69] Martinant J. P. Billot A. Bouguennec A. Charmet G. Saulnier L. Branlard G. (1999). Genetic and environmental variations in water-extractable arabinoxylans content and flour extract viscosity. *J. Cereal Sci.* 30 45–48. 10.1006/jcrs.1998.0259

[B70] Martis M. M. Zhou R. Haseneyer G. Schmutzer T. Vrána J. Kubaláková M. (2013). Reticulate evolution of the rye genome. *Plant Cell* 25 3685–3698. 10.1105/tpc.113.114553 24104565 PMC3877785

[B71] Mayer K. F. X. Martis M. Hedley P. E. Šimková H. Liu H. Morris J. A. (2011). Unlocking the barley genome by chromosomal and comparative genomics. *Plant Cell* 23 1249–1263. 10.1105/tpc.110.082537 21467582 PMC3101540

[B73] McIntosh G. H. Whyte J. McArthur R. Nestel P. J. (1991). Barley and wheat foods – Influence on plasma-cholesterol concentrations in hypercholesterolemic men. *Am. J. Clin. Nutr.* 53 1205–1209. 1850576 10.1093/ajcn/53.5.1205

[B74] Medouri A. Bellil I. Khelifi D. (2015). Polymorphism at high molecular weight glutenin subunits and morphological diversity of *Aegilops geniculata* Roth collected in Algeria. *Cereal Res. Commun.* 43 272–283. 10.1556/CRC.2014.0042

[B75] Mikó P. Löschenberger F. Hiltbrunner J. Aebi R. Megyeri M. Kovács G. (2014). Comparison of bread wheat varieties with different breeding origin under organic and low input management. *Euphytica* 199 69–80. 10.1016/j.dib.2016.04.065

[B76] Miller S. S. Wood P. J. Pietrzak L. N. Fulcher R. G. (1993). Mixed linkage beta glucans, protein content, and kernel weight in Avena species. *Cereal Chem.* 70 231–233.

[B77] Mitchell R. A. C. Dupree P. Shewry P. R. (2007). A novel bioinformatics approach identifies candidate genes for the synthesis and feruloylation of arabinoxylan. *Plant Physiol.* 144 43–53. 10.1104/pp.106.094995 17351055 PMC1913792

[B78] Molina-Cano J. L. Moralejo M. Elia M. Munoz P. Russell J. R. Perez-Vendrell A. M. (2007). QTL analysis of a cross between European and North American malting barleys reveals a putative candidate gene for beta-glucan content on chromosome 1H. *Mol. Breed.* 19 275–284. 10.1007/s11032-006-9075-5

[B79] Molnár I. Gáspár L. Sárvári É Dulai S. Hoffmann B. Molnár-Láng M. (2004). Physiological and morphological responses to water stress in *Aegilops biuncialis* and *Triticum aestivum* genotypes with differing tolerance to drought. *Funct. Plant Biol.* 31 1149–1159. 10.1071/FP03143 32688982

[B80] Molnár I. Vrána J. Burešová V. Cápal P. Farkas A. Darkó É (2016). Dissecting the U, M, S and C genomes of wild relatives of bread wheat (Aegilops spp.) into chromosomes and exploring their synteny with wheat. *Plant J.* 88 452–467. 10.1111/tpj.13266 27402341

[B81] Molnár-Láng M. Linc G. Nagy E. D. Schneider A. Molnár I. (2002). Molecular cytogenetic analysis of wheat-alien hybrids and derivatives. *Acta Agron. Hung.* 50 303–311. 10.1556/AAgr.50.2002.3.8

[B82] Molnár-Láng M. Linc G. Sutka J. (1996). Transfer of the recessive crossability allele kr1 from Chinese Spring into the winter wheat variety Martonvásári 9. *Euphytica* 90 301–305. 10.1007/BF00027480

[B83] Morales-Ortega A. Carvajal-Millan E. Lopez-Franco Y. Rascon-Chu A. Lizardi- Mendoza J. Torres-Chavez P. (2013). Characterization of water extractable arabinoxylans from a spring wheat flour: rheological properties and microstructure. *Molecules* 18 8417–8428. 10.3390/molecules18078417 23863779 PMC6269794

[B84] MSZ 6367/4-86 (1987). *Edible, Fodder and Industrial Seeds Husked Products. Determination of Test Weight, GradeThousand Kernel Weight and Classification.* Budapest: Hungarian Standards Institution Available at: www.mszt.hu

[B85] Nemeth C. Freeman J. Jones H. D. Sparks C. Pellny T. K. Wilkinson M. D. (2010). Downregulation of the CSLF6 gene results in decreased (1,3;1,4)-β-D-glucan in endosperm of wheat. *Plant Physiol.* 152 1209–1218. 10.1104/pp.109.151712 20089768 PMC2832239

[B86] Noort M. W. J. van Haaster D. Hemery Y. Schols H. A. Hamer R. J. (2010). The effect of particle size of wheat bran fractions on bread quality - Evidence for fibre protein interactions. *J. Cereal Sci.* 52 59–64. 10.1016/j.jcs.2010.03.003

[B87] Ordaz-Ortiz J. J. Devaux M. F. Saulnier L. (2005). Classification of wheat varieties based on structural features of arabinoxylans as revealed by endoxylanase treatment of flour and grain. *J. Agric. Food Chem.* 53 8349–8356. 10.1021/jf050755v 16218687

[B88] Ordaz-Ortiz J. J. Guillon F. Tranquet O. Dervilly-Pinel G. Tran V. Saulnier L. (2004). Specificity of monoclonal antibodies generated against arabinoxylans of cereal grains. *Carbohydr. Polym.* 57 425–433. 10.1016/j.carbpol.2004.05.016

[B89] Ordaz-Ortiz J. J. Saulnier L. (2005). Structural variability of arabinoxylans from wheat flour. Comparison of water-extractable and xylanase-extractable arabinoxylans. *J. Cereal Sci.* 42 119–125. 10.1016/j.jcs.2004.02.004

[B90] Payne P. I. (1987). Genetics of wheat storage proteins and the effect of allelic variation on bread-making quality. *Annu. Rev. Plant Physiol.* 38 141–153. 10.1146/annurev.pp.38.060187.001041

[B91] Pellny T. K. Lovegrove A. Freeman J. Tosi P. Love C. G. Knox J. P. (2012). Cell walls of developing wheat starchy endosperm: comparison of composition and RNA-Seq transcriptome. *Plant Physiol.* 158 612–627. 10.1104/pp.111.189191 22123899 PMC3271754

[B92] Perlin A. S. (1951). Structure of the soluble pentosans of wheat flours. *Cereal Chem.* 28 382–393.

[B93] Pirgozliev V. Rose S. P. Pellny T. Amerah A. M. Wickramasinghe M. Ulker M. (2015). Energy utilization and growth performance of chickens fed novel wheat inbred lines selected for different pentosan levels with and without xylanase supplementation. *Poult. Sci.* 94 232–239. 10.3382/ps/peu059 25595480 PMC4988544

[B94] Quraishi U. M. Murat F. Abrouk M. Pont C. Confolent C. Oury F. X. (2011). Combined meta-genomics analyses unravel candidate genes for the grain dietary fiber content in bread wheat (*Triticum aestivum* L.). *Funct. Integr. Genomics* 11 71–83. 10.1007/s10142-010-0183-2 20697765

[B95] Rakszegi M. Lang L. Bedo Z. Shewry P. R. (2008). Composition and end-use quality of 150 wheat lines selected for the HEALTHGRAIN diversity screen. *J. Agric. Food Chem.* 56 9750–9757. 10.1021/jf8009359 18921975

[B96] Rawat N. Tiwari V. K. Singh N. Randhawa G. S. Singh K. Chhuneja P. (2009). Evaluation and utilization of *Aegilops* and wild *Triticum* species for enhancing iron and zinc content in wheat. *Genet. Resour. Crop Evol.* 56 53–64. 10.1007/s10722-008-9344-8

[B97] Rekika D. Monneveux P. Havaux M. (1997). The in vivo tolerance of photosynthetic membranes to high and low temperatures in cultivated and wild wheats of the Triticum and *Aegilops genera*. *J. Plant Physiol.* 150 734–738. 10.1016/S0176-1617(97)80291-X

[B98] Renard C. M. G. C. Rouau X. Thibault J. F. (1990). Structure and properties of water-soluble pentosans from wheat flour. *Sci. Aliments* 10 283–292.

[B99] Rey E. Molnár I. Doležel J. (2015). “Genomics of wild relatives and alien introgressions,” in *Alien Introgression in Wheat*, eds Molnár-Láng M. Ceoloni C. Doležel J. (Cham: Springer International Publishing), 347–381.

[B100] Saulnier L. Robert P. Grintchenko M. Jamme F. Bouchet B. Guillon F. (2009). Wheat endosperm cell walls: spatial heterogeneity of polysaccharide structure and composition using micro-scale enzymatic fingerprinting and FT-IR microspectroscopy. *J. Cereal Sci.* 50 312–317. 10.1016/j.jcs.2009.05.003

[B101] Saulnier L. Sado P. E. Branlard G. Charmet G. Guillon F. (2007). Wheat arabinoxylans: exploiting variation in amount and composition to develop enhanced varieties. *J. Cereal Sci.* 46 261–281. 10.1016/j.jcs.2007.06.014

[B102] Schneider A. Linc G. Molnár I. Molnár-Láng M. (2005). Molecular cytogenetic characterization of *Aegilops biuncialis* and its use for the identification of five derived wheat-*Aegilops biuncialis* disomic addition lines. *Genome* 48 1070–1082. 10.1139/g05-062 16391676

[B103] Schneider A. Molnár I. Molnár-Láng M. (2008). Utilisation of *Aegilops* (goatgrass) species to widen the genetic diversity of cultivated wheat. *Euphytica* 163 1–19. 10.1007/s10681-007-9624-y

[B104] Schooneveld-Bergmans M. E. F. Beldman G. Voragen A. G. J. (1999). Structural features of (glucurono) arabinoxylans extracted from wheat bran by barium hydroxide. *J. Cereal Sci.* 29 63–75. 10.1006/jcrs.1998.0222

[B105] Schreiber M. Wright F. MacKenzie K. Hedley P. E. Schwerdt J. G. Little A. (2014). The barley genome sequence assembly reveals three additional members of the *CslF* (1,3;1,4)-β-Glucan synthase gene family. *PLoS ONE* 9:e90888 10.1371/journal.pone.0090888 PMC3940952 24595438

[B106] Shewry P. R. D’Ovidio R. Lafiandra D. Jenkins J. A. Mills E. N. C. Bekes F. (2009). “Wheat grain proteins,” in *Wheat: Chemistry and Technology*, eds Khan K. Shewry P. R. (St. Paul, MN: AACC), 223–298. 10.1094/9781891127557.008

[B107] Shewry P. R. Freeman J. Wilkinson M. Pellny T. Mitchell R. A. C. (2010a). “Challenges and opportunities for using wheat for biofuel production,” in *Energy Crops*, eds Halford N. Karp A. (London: Royal Society of Chemistry), 13–26.

[B108] Shewry P. R. Piironen V. Lampi A. M. Edelmann M. Kariluoto S. Nurmi T. (2010b). The HEALTHGRAIN wheat diversity screen: effects of genotype and environment on phytochemicals and dietary fiber components. *J. Agric. Food Chem.* 58 9291–9298. 10.1021/jf100039b 20438061

[B109] Shewry P. R. Saulnier L. Guillon F. Gebruers K. Courtin C. Delcour J. (2010c). “Improving the benefits of wheat as a source of dietary fibre,” in *Dietary Fibre: New Frontiers for Food and Health*, eds van der Kamp J. W. Jones J. McCleary B. Topping D. (Wageningen: Wageningen Academic Publishers), 65–78.

[B110] Shewry P. R. Halford N. G. Lafiandra D. (2003a). “The genetics of wheat gluten proteins,” in *Advances in Genetics* Vol. 49 eds Hall J. C. Dunlap J. C. Friedman T. (Cambridge, MA: Academic Press), 111–184. 10.1016/s0065-2660(03)01003-4 12779252

[B111] Shewry P. R. Halford N. G. Tatham A. S. Popineau Y. Lafiandra D. Belton P. (2003b). The high molecular weight subunits of wheat glutenin and their role in determining wheat processing properties. *Adv. Food Nutr. Res.* 45 221–302. 10.1016/S1043-4526(03)45006-7 12402682

[B112] Shewry P. R. Li L. Piironen V. Lampi A. M. Nystrom L. Rakszegi M. (2008). Phytochemicals and fiber components in oat varieties in the HEALTHGRAIN diversity screen. *J. Agric. Food Chem.* 56 9777–9784. 10.1021/jf801880d 18921980

[B113] Shewry P. R. Tatham A. S. Forde J. Kreis M. Miflin B. J. (1986). The classification and nomenclature of wheat gluten proteins: a reassessment. *J. Cereal Sci.* 4 97–106. 10.1016/S0733-5210(86)80012-1

[B114] Shu X. L. Rasmussen S. K. (2014). Quantification of amylose, amylopectin, and beta-glucan in search for genes controlling the three major quality traits in barley by genome-wide association studies. *Front. Plant Sci.* 5:197 10.3389/fpls.2014.00197 PMC4030205 24860587

[B115] Šimková H. Svensson J. T. Condamine P. Hřibová E. Suchánková P. Bhat P. R. (2008). Coupling amplified DNA from flow-sorted chromosomes to high-density SNP mapping in barley. *BMC Genomics* 9:294 10.1186/1471-2164-9-294 PMC2453526 18565235

[B116] Skendi A. Papageorgiou M. Biliaderis C. G. (2009). Effect of barley β-glucan molecular size and level on wheat dough rheological properties. *J. Food Eng.* 91 594–601. 10.1016/j.jfoodeng.2008.10.009

[B117] Storsley J. M. Izydorczyk M. S. You S. Biliaderis C. G. Rossnagel B. (2003). Structure and physicochemical properties of b-glucans and arabinoxylans isolated from hull-less barley. *Food Hydrocoll.* 17 831–844. 10.1016/S0268-005X(03)00104-8

[B118] Symons L. J. Brennan C. S. (2004). The influence of a (1-3, 1-4)-β-D-glucan rich fraction on the physico-chemical properties and in vitro reducing sugar release of white wheat breads. *J. Food Sci.* 69 463–467. 10.1111/j.1365-2621.2004.tb10989.x

[B119] Taketa S. Yuo T. Tonooka T. Tsumuraya Y. Inagaki Y. Haruyama N. (2012). Functional characterization of barley betaglucanless mutants demonstrates a unique role for CslF6 in (1,3;1,4)-β-D-glucan biosynthesis. *J. Exp. Bot.* 63 381–392. 10.1093/jxb/err285 21940720 PMC3245474

[B120] The International Wheat Genome Sequencing Consortium [IWGSC] (2014). A chromosome-based draft sequence of the hexaploid bread wheat (*Triticum aestivum*) genome. *Science* 345:1251788 10.1126/science.1251788 25035500

[B121] Tighe P. Duthie G. Vaughan N. Brittenden J. Simpson W. G. Duthie S. (2010). Effect of increased consumption of whole-grain foods on blood pressure and other cardiovascular risk markers in healthy middle-aged persons: a randomized controlled trial. *Am. J. Clin. Nutr.* 92 733–740. 10.3945/ajcn.2010.29417 20685951

[B122] Tiwari V. K. Wang S. Danilova T. Koo D. H. Vrána J. Kubaláková M. (2015). Exploring the tertiary gene pool of bread wheat: sequence assembly and analysis of chromosome 5Mg of *Aegilops geniculata*. *Plant J.* 84 733–746. 10.1111/tpj.13036 26408103

[B123] Toole G. A. Le Gall G. Colquhoun I. J. Nemeth C. Saulnier L. Lovegrove A. (2010). Temporal and spatial changes in cell wall composition in developing grains of wheat cv. *Hereward. Planta* 232 677–689. 10.1007/s00425-010-1199-5 20549231

[B124] Vaikousi H. Biliaderis C. G. Izydorczyk M. S. (2004). Solution flow behaviour and gelling properties of water- soluble barley (1-3, 1-4)-beta-glucans varying in molecular size. *J. Cereal Sci.* 39 119–137. 10.1016/j.jcs.2003.09.001

[B125] van Slageren M. W. (1994). *Wild Wheats: a Monograph of Aegilops L. and Amblyopyrum (Jaub and Spach) Eig (Poaceae).* Wageningen: Wageningen Agricultural University Papers.

[B126] Wan Y. Poole R. L. Huttly A. K. Toscano-Underwood C. Feeney K. Welham S. (2008). Transcriptome analysis of grain development in hexaploid wheat. *BMC Genomics* 9:121 10.1186/1471-2164-9-121 PMC2292175 18325108

[B127] Wang S. L. Chen D. Guo G. F. Zhang T. Jiang S. S. Shen X. X. (2012). Molecular characterization of LMW-GS genes from C, N, U and S-s genomes among Aegilops species. *Cereal Res. Commun.* 40 542–551. 10.1007/s00122-010-1354-1

[B128] Wang Y. J. Wang C. Y. Zhang H. Li H. Liu X. L. Ji W. Q. (2015). Identification and evaluation of disease resistance and HMW-GS composition of *Aegilops geniculata* Roth. *Genet. Resour. Crop Evol.* 62 1085–1093. 10.1007/s10722-015-0217-7

[B129] Ward J. L. Poutanen K. Gebruers K. Piironen V. Lampi A. M. Nystrom L. (2008). The HEALTHGRAIN cereal diversity screen: concept, results and prospects. *J. Agric. Food Chem.* 56 9699–9709. 10.1021/jf8009574 18921969

[B130] Wood P. J. (2007). Cereal β-glucans in diet and health. *J. Cereal Sci.* 46 230–238. 10.1016/j.jcs.2007.06.012

[B131] Wood P. J. Weisz J. Beer M. U. Newman C. W. Newman R. K. (2003). Structure of (1-3)(1-4)-β-D-glucan in waxy and nonwaxy barley. *Cereal Chem.* 80 329–332. 10.1094/CCHEM.2003.80.3.329

[B132] Zaharieva M. Gaulin E. Havaux M. Acevedo E. Monneveux P. (2001a). Drought and heat responses in the wild wheat relative *Aegilops geniculata* Roth. *Crop Sci.* 41 1321–1329. 10.2135/cropsci2001.4141321x

[B133] Zaharieva M. Monneveux P. Henry M. Rivoal R. Valkoun J. Nachit M. M. (2001b). Evaluation of a collection of wild wheat relative *Aegilops geniculata* Roth and identification of potential sources for useful traits. *Euphytica* 119 33–38. 10.1023/A:1017500728227

[B134] Zeng W. Jiang N. Nadella R. Killen T. L. Nadella V. Faik A. A. (2010). Glucurono(arabino)xylan synthase complex from wheat contains members of the GT43, GT47, and GT75 families and functions cooperatively. *Plant Physiol.* 154 78–97. 10.1104/pp.110.159749 20631319 PMC2938142

[B135] Zimin A. V. Marçais G. Puiu D. Roberts M. Salzberg S. L. Yorke J. A. (2013). The MaSuRCA genome assembler. *Bioinformatics* 29 2669–2677. 10.1093/bioinformatics/btt476 23990416 PMC3799473

