# Supplementary figures and images for: Addition of Aegilops U and M Chromosomes Affects Protein and Dietary Fiber Content of Wholemeal Wheat Flour
Source: Front Plant Sci. 2017 Sep 6;8:1529. doi: 10.3389/fpls.2017.01529 (PMC5592229; doi:10.3389/fpls.2017.01529)

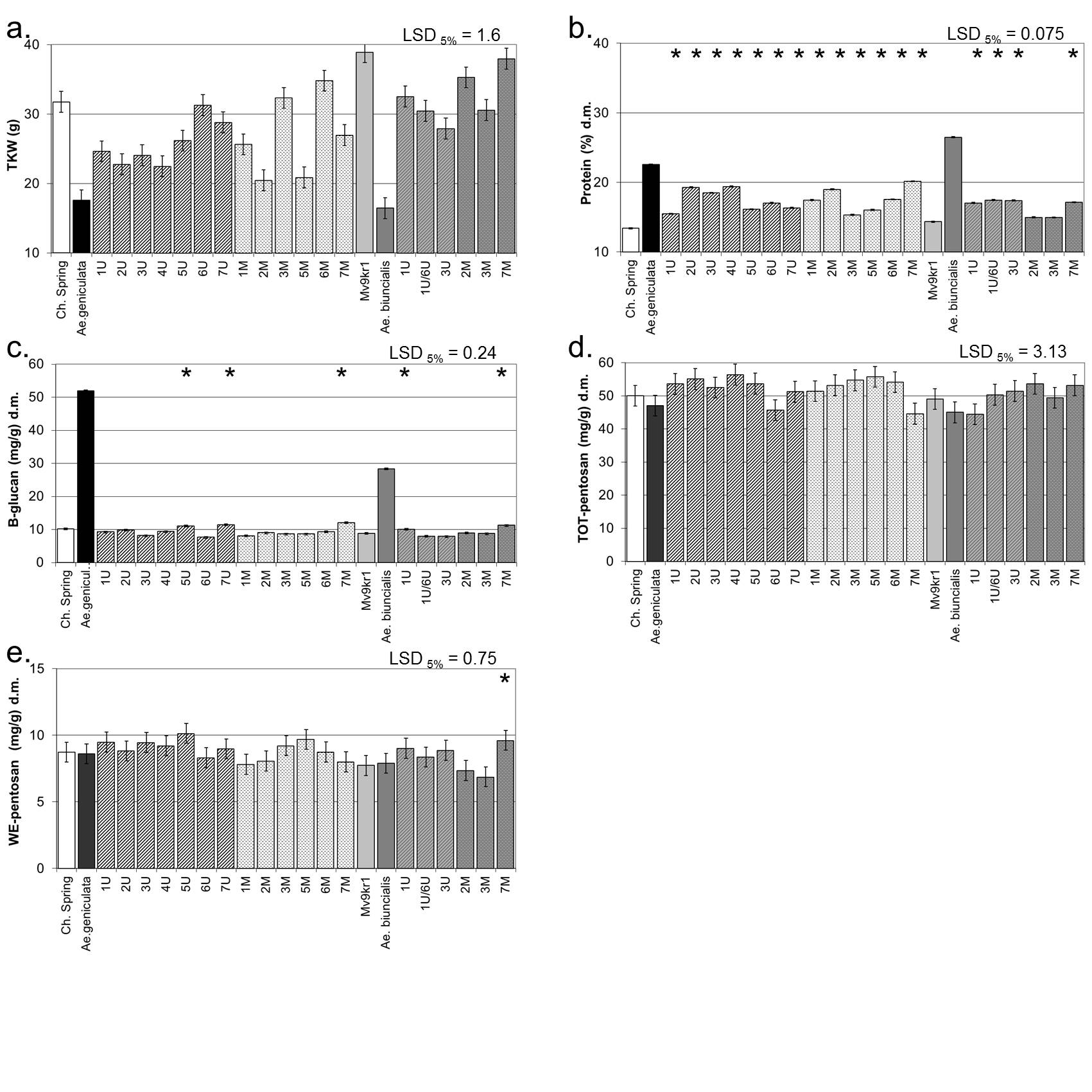

Supplement: Supplementary file 2 [file Image_2.JPEG]
