# Supplementary material for: Addition of Aegilops U and M Chromosomes Affects Protein and Dietary Fiber Content of Wholemeal Wheat Flour
Source: Front Plant Sci. 2017 Sep 6;8:1529. doi: 10.3389/fpls.2017.01529 (PMC5592229; doi:10.3389/fpls.2017.01529)
Supplement: Supplementary file 3 [file Table_1.DOCX]

Supplementary Table 1. Blast search on selected protein alleles of *Triticum aestivum* in the *Aegilops umbellulata* genome

| **Query** |  |  | ***T. aestivum*** | **BLASTn results on Ae. umbellulata sequences** | ***Ae. umbellulata*** |  |  |  |  |  |
| --- | --- | --- | --- | --- | --- | --- | --- | --- | --- | --- |
| **HMW glutenins** | **Gene** | **GenBank Acc. No.*** | **Chromosome** | **Scaffold** | **Chromosome** | **Score value** | **E value** | **Percentage identity** | **Match length** | **Reference** |
| 1Ax1 | Glu-1Ax1 | X61009* | 1A | jcf7180008768359_1u | 1U | 2435.83 | 0.00 × 10^+00^ | 78.54 | 2134 | Halford et al. 1992 |
| 1Ax2* | Glu-1Ax2 | M22208.2* | 1A | jcf7180008768359_1u | 1U | 5887.48 | 0.00 × 10^+00^ | 83.35 | 4466 | Anderson and Greene 1989 |
| 1Bx7 | Glu-B1-1b | X13927.3* | 1B | jcf7180008768359_1u | 1U | 5274.34 | 0.00 × 10^+00^ | 80.60 | 4332 | Anderson and Greene 1989 |
| 1Dx5 | Glu-1D-1d | X12928.5* | 1D | jcf7180008768359_1u | 1U | 7753.97 | 0.00 × 10^+00^ | 91.95 | 4853 | Anderson and Greene 1989 |
| 1Dy10 | Glu-D1-2b | X12929.2 | 1D | jcf7180008765612_1u | 1U | 4745.95 | 0.00 × 10^+00^ | 86.01 | 3438 | Anderson et al. 1989 |
| 1Dy12 |  | X03041.1 | 1D | jcf7180008765612_1u | 1U | 3716.23 | 0.00 × 10^+00^ | 85.66 | 2693 | Thompson et al. 1985 |
| 1Ux (Au) | Glu-1Ux | AF476961.1 | - | jcf7180008768359_1u | 1U | 3297.84 | 0.00 × 10^+00^ | 85.36 | 2390 | Liu et al. 2003 |
| 1Uy (Au) | Glu-1Uy | AF476962.1 | - | jcf7180008765612_1u | 1U | 1718.09 | 0.00 × 10^+00^ | 79.38 | 1455 | Liu et al. 2003 |
| **LMW glutenins** |  |  |  |  |  |  |  |  |  |  |
|  |  | AB062868.1 | 1D | jcf7180008691789_1u | 1U | 1622.51 | 0.00 × 10^+00^ | 91.39 | 1040 | Ikeda et al. 2002 |
|  |  | AB062872.1 | 1D | jcf7180008691789_1u | 1U | 1772.19 | 0.00 × 10^+00^ | 94.61 | 1071 | Ikeda et al. 2002 |
|  | Glu-B3b | JX163862.1 | 1B | jcf7180008725136_1u | 1U | 1811.87 | 0.00 × 10^+00^ | 90.93 | 1173 | Zeng et al. 2010 |
|  | Glu-D3 | HM055909.1 | 1DS | jcf7180008691789_1u | 1U | 1379.0 | 0.00 × 10^+00^ | 93.60 | 906 | Anders et al. 2012 |
|  |  | Y17845.1 | 1BS | jcf7180008725136_1u | 1U | 1645.96 | 0.00 × 10^+00^ | 91.07 | 1153 | Shewry et al. 2009 |
|  |  | U86026.1 | 1DS | jcf7180008691789_1u | 1U | 1893.02 | 0.00 × 10^+00^ | 90.37 | 1381 | Shewry et al. 2009 |
|  |  | X13306.1 | 1DS | jcf7180008725136_1u | 1U | 1040.03 | 0.00 × 10^+00^ | 81.43 | 1066 | Shewry et al. 2009 |
|  |  | AB062875.1 | 1DS | jcf7180008691789_1u | 1U | 1270.86 | 0.00 × 10^+00^ | 82.92 | 1183 | Shewry et al. 2009 |
|  |  | U86028.1 | 1DS | jcf7180008691789_1u | 1U | 2057.13 | 0.00 × 10^+00^ | 94.68 | 1316 | Shewry et al. 2009 |
|  |  | X07747.1 | 1AS | jcf7180008725136_1u | 1U | 1860.56 | 0.00 × 10^+00^ | 80.41 | 1955 | Shewry et al. 2009 |
|  |  | AB062873.1 | 1DS | jcf7180008691789_1u | 1U | 1552.18 | 0.00 × 10^+00^ | 90.41 | 1126 | Anders et al. 2012 |
| **γ-gliadins** | **(Group/Pattern/Subgroup)** |  |  |  |  |  |  |  |  |  |
|  | C10/C10-P1/SG-1 | AJ937838.1 | 1DS | jcf7180008681874_1u | 1U | 450.32 | 5.80 × 10-124 | 71.65 | 889 | Qi et al. 2009 |
|  | C9/C9-P2/SG-2 | AF234646.1 | 1DS | jcf7180008773847_1u | 1U | 1662.19 | 0.00 × 10^+00^ | 87.20 | 1367 | Qi et al. 2009 |
|  | C9/C9-P3/SG-3 | FJ006638.1 | 1DS | jcf7180008733211_1u | 1U | 1000.35 | 0.00 × 10^+00^ | 86.81 | 819 | Qi et al. 2009 |
|  | C9/C9-P4/SG-4 | FJ006605.1 | 1DS | jcf7180008771872_1u | 1U | 1490.87 | 0.00 × 10^+00^ | 94.39 | 945 | Qi et al. 2009 |
|  | C9/C9-P4/SG-6 | AF234647.1 | 1BS | jcf7180008773847_1u | 1U | 2789.29 | 0.00 × 10^+00^ | 88.12 | 2180 | Qi et al. 2009 |
|  | C9/C9-P4/SG-7 | FJ006596.1 | 1DS | jcf7180008716048_1u | 1U | 1357.42 | 0.00 × 10^+00^ | 88.94 | 1040 | Qi et al. 2009 |
|  | C8/C8-P5 /SG-8 | AF175312.1 | 1DS | jcf7180008681874_1u | 1U | 1068.88 | 0.00 × 10^+00^ | 85.24 | 928 | Qi et al. 2009 |
|  | C8/C8-P5 /SG-9 | AF120267.1 | 1DS | jcf7180008681874_1u | 1U | 1335.78 | 0.00 × 10^+00^ | 89.64 | 985 | Qi et al. 2009 |
|  | C8/C8-P5 /SG-12 | AF234649.1 | 1DS | jcf7180008773847_1u | 1U | 2244.68 | 0.00 × 10^+00^ | 84.07 | 2097 | Qi et al. 2009 |
|  | C8/C8-P5 /SG-13 | AF234643.1 | 1AS | jcf7180008733211_1u | 1U | 1067.08 | 0.00 × 10^+00^ | 83.38 | 975 | Qi et al. 2009 |
|  | C7/C7-P6 /SG-14 | AJ416336.1 | 1DS | jcf7180008681874_1u | 1U | 1144.62 | 0.00 × 10^+00^ | 88.61 | 869 | Qi et al. 2009 |
|  | C7/C7-P7 /SG-15 | M16064.1 | 1DS | jcf7180008681874_1u | 1U | 1701.86 | 0.00 × 10^+00^ | 86.93 | 1400 | Qi et al. 2009 |
| **α-gliadins** |  |  |  |  |  |  |  |  |  |  |
|  |  | AJ133612.1 | 6AS | jcf7180008773718_1u | 1U | 1106.75 | 0.00 × 10^+00^ | 86.05 | 889 | Shewry et al. 2009 |
|  |  | DQ166377.1 | 6AS | jcf7180008773718_1u | 1U | 1195.12 | 0.00 × 10^+00^ | 89.03 | 893 | Shewry et al. 2009 |
|  |  | K03074.1 | 2BS | jcf7180008773718_1u | 1U | 2062.54 | 0.00 × 10^+00^ | 87.02 | 1641 | Shewry et al. 2009 |
|  |  | M11075.1 | 6AS | jcf7180008773718_1u | 1U | 1582.84 | 0.00 × 10^+00^ | 91.03 | 1115 | Shewry et al. 2009 |
|  |  |  |  | scf7180031650559_2u | 2U | 1543.17 | 0.00 × 10^+00^ | 88.39 | 1180 | Wheat könyv |
|  |  | U08287.1 | 6AS | jcf7180008773718_1u | 1U | 2316.81 | 0.00 × 10^+00^ | 90.41 | 1658 | Shewry et al. 2009 |
|  |  |  |  | scf7180011455011_3u | 3U | 1849.74 | 0.00 × 10^+00^ | 92.53 | 1258 | Shewry et al. 2009 |
|  |  | X01130.1 | 6AS | jcf7180008773718_1u | 1U | 2293.37 | 0.00 × 10^+00^ | 90.22 | 1656 | Shewry et al. 2009 |
|  |  |  |  | scf7180011455011_3u | 3U | 1819.08 | 0.00 × 10^+00^ | 91.98 | 1259 | Shewry et al. 2009 |
|  |  | U50984.1 | 6AS | jcf7180008773718_1u | 1U | 2154.51 | 0.00 × 10^+00^ | 89.46 | 1613 | Shewry et al. 2009 |
|  |  |  |  | scf7180011455011_3u | 3U | 1721.70 | 0.00 × 10^+00^ | 91.76 | 1214 | Shewry et al. 2009 |
|  |  | X02539.1 | 6AS | jcf7180008773718_1u | 1U | 2284.35 | 0.00 × 10^+00^ | 91.10 | 1607 | Shewry et al. 2009 |
|  |  |  |  | scf7180011455011_3u | 3U | 1810.07 | 0.00 × 10^+00^ | 93.22 | 1210 | Shewry et al. 2009 |

List of *Triticum aestivum* alleles selected from ’Wheat: Chemistry and Technology, Eds. Khan K. and Shewry P.R., 2009.’

*: NCBI (https://www.ncbi.nlm.nih.gov/)

Anderson, O.D. and Greene, F.C. (1989). The characterization and comparative analysis of high-molecular-weight glutenin genes from genomes A and B of a hexaploid bread wheat. *Theor. Appl. Genet*. 77, 689-700.

Anderson, O.D., Greene, F.C., Yip, R.E., Halford, N.G., Shewry, P.R. and Malpica-Romero, J.M. (1989). Nucleotide sequences of the two high-molecular-weight glutenin genes from the D-genome of a hexaploid bread wheat, Triticum aestivum L. cv Cheyenne. *Nucleic Acids Res*. 17, 461-462.

Halford, N.G., Field, J.M., Blair, H., Urwin, P., Moore, K., Robert, L., Thompson, R., Flavell, R.B., Tatham, A.S., Shewry, P.R. (1992). Analysis of HMW glutenin subunits encoded by chromosome 1A of bread wheat (*Triticum aestivum* L.) indicates quantitative effects on grain quality. *Theor. Appl. Genet*. 83, 373-378.

Ikeda, T.M., Nagamine, T., Fukuoka, H., Yano, H. (2002). Identification of new low-molecular-weight glutenin subunit genes in wheat. *Theor. Appl. Genet*. 104, 680-687.

Liu, Z., Yan, Z., Wan, Y., Liu, K., Zheng, Y., Wang, D. (2003). Analysis of HMW glutenin subunits and their coding sequences in two diploid Aegilops species. *Theor. Appl*. *Genet.* 106, 1368-1378.

Piston, F., Dorado, G., Martin, A., Barro, F. (2006). Cloning of nine gamma-gliadin mRNAs (cDNAs) from wheat and the molecular characterization of comparative transcript levels of gamma-gliadin subclasses. *J. Cereal Sci*. 43, 120-128.

Qi, P.F., Wei, Y.M., Ouellet, T., Chen, Q., Tan, X., Zheng, Y.L. (2009). The gamma-gliadin multigene family in common wheat (*Triticum aestivum*) and its closely related species. *BMC Genomics* 10, 168.

Shewry, P.R., D’Ovidio R., Lafiandra, D., Jenkins, J.A., Mills E.N.C., Békés, F. (2009) Wheat Grain Proteins. In: Wheat Chemistry and Technology (Eds.: Khan K., Shewry, P.R.). AACC International Inc., p. 223-298.

Thompson, R.D., Bartels, D., Harberd, N.P. (1985) Nucleotide sequence of a gene from chromosome 1D of wheat encoding a HMW-glutenin subunit. *Nucleic Acids Res*. 13, 6833-6846.
