# Supplementary material for: Addition of Aegilops U and M Chromosomes Affects Protein and Dietary Fiber Content of Wholemeal Wheat Flour
Source: Front Plant Sci. 2017 Sep 6;8:1529. doi: 10.3389/fpls.2017.01529 (PMC5592229; doi:10.3389/fpls.2017.01529)
Supplement: Supplementary file 4 [file Table_2.DOCX]

Supplementary Table 2. Blast search on selected β-glucan alleles of *Oryza sativa and Hordeum vulgare* L. in the *Aegilops umbellulata* genome

| **Query** |  |  | ***T. aestivum*** | **BLASTn results *on Ae. umbellulata* sequences** | ***Ae. umbellulata*** |  |  |  |  |  |
| --- | --- | --- | --- | --- | --- | --- | --- | --- | --- | --- |
| **Organism** | **Gene** | **GenBank Acc. No.*** | **Chromosome**  **(wheat)** | **Scaffold** | **Chromosome** | **Score value** | **E value** | **Percentage identity** | **Match length** | **Reference** |
| Rice | *OsCslF1* | AF432502.1 | 2AS, 2BS | scf7180031527412_2u | 2U | 1656.78 | 0.00 × 10^+00^ | 81.75 | 1682 | - |
|  | *OsCslF2* | AF432503.1 | 2BL | scf7180031527412_2u | 2U | 1651.37 | 0.00 × 10^+00^ | 82.69 | 1618 | - |
| Barley | *HvCslF3* | EU267179.1 | 2AS, 2BS, 2DS | scf7180031568502_2u | 2U | 2803.72 | 0.00 × 10^+00^ | 95.50 | 1676 | Burton et al. 2008 |
|  | *HvCslF4* | EU267180.1 | 2AS, 2BS | scf7180031527412_2u | 2U | 2610.76 | 0.00 × 10^+00^ | 93.12 | 1625 | Burton et al. 2008 |
|  | *HvCslF6* | EU267181.1 | 7DL | scf7180014342612_7ul | 7UL | 2975.04 | 0.00 × 10^+00^ | 94.61 | 1807 | Burton et al. 2008 |
|  | *HvCslF7* | EU267182.1 | 5BL | scf7180016161383_5u | 5U | 3409.65 | 0.00 × 10^+00^ | 92.43 | 2337 | Burton et al. 2008 |
|  | *HVCslF8* | EU267183.1 | 2AS, 2BS, 2DS | scf7180031483586_2u | 2U | 3320 | 0.00 × 10^+00^ | 95.08 | 1889 | Burton et al. 2008 |
|  | *HVCslF9* | EU267184.1 | 1AS, 1BS, 1DS | jcf7180008683045_1u | 1U | 2556.66 | 0.00 × 10^+00^ | 92.45 | 1603 | Burton et al. 2008 |
|  | *HvCslF10* | EU267185.1 | 2AS, 2BS, 2DS | scf7180031569918_2u | 2U | 2612.56 | 0.00 × 10^+00^ | 93.87 | 1713 | Burton et al. 2008 |
|  | *HvCslF11* | - | 7DL, 7BL | jcf7180007988693_6u | 6U | 205450 | 0.00 × 10^+00^ | 94.3 | 1609 | Schreiber et al. 2014 |
|  | *HvCslF12* | - | 2AS, 2BS, 2DS | scf7180031568183_2u | 2U | 2564.27 | 0.00 × 10^+00^ | 95.3 | 1625 | Schreiber et al. 2014 |
|  | *HvCslF13* | - | 2AL, 2BL | scf7180031527412_2u | 2U | 767.48 | 0.00 × 10^+00^ | 89.5 | 607 | Schreiber et al. 2014 |
|  | *HvCslH1* | - | 2AL, 2BL, 2AL | scf7180031648903_2u | 2U | 824.72 | 0.00 × 10^+00^ | 93.4 | 559 | Schreiber et al. 2014 |

*: NCBI (https://www.ncbi.nlm.nih.gov/)

Burton, R.A., Jobling, S.A., Harvey, A.J., Shirley, N.J., Mather, D.E., Bacic, A., Fincher,G.B. (2008). The genetics and transcriptional profiles of the cellulose synthase-like HvCslF gene family in barley. *Journal of Plant Physiol.* 146, 1821-1833.

Schreiber, M., Wright, F., MacKenzie, K., Hedley, P.E., Schwerdt, J.G., et al. (2014). The barley genome sequence assembly reveals three additional members of the CslF (1,3;1,4)-b -glucan synthase gene family*. PLoS ONE* 9(3), e90888. doi:10.1371/journal.pone.0090888
