# Supplementary material for: Addition of Aegilops U and M Chromosomes Affects Protein and Dietary Fiber Content of Wholemeal Wheat Flour
Source: Front Plant Sci. 2017 Sep 6;8:1529. doi: 10.3389/fpls.2017.01529 (PMC5592229; doi:10.3389/fpls.2017.01529)
Supplement: Supplementary file 5 [file Table_3.DOCX]

Supplementary Table 3. BLASTn search on recently discovered wheat (*Triticum aestivum*) arabinoxylan biosynthesis-related genes in *Aegilops umbellulata* chromosome survey sequences

| **Query** |  |  | ***T. aestivum*** | **BLASTn results on Ae. umbellulata sequences** | ***Ae. umbellulata*** |  |  |  |  |  |
| --- | --- | --- | --- | --- | --- | --- | --- | --- | --- | --- |
| **Organism** | **Gene** | **GenBank Acc. No.*** | **Chromosome** | **Scaffold** | **Chromosome** | **Score value** | **E value** | **Percentage identity** | **Match length** | **Reference** |
| wheat | *TaGT43-2D* | HF913567.1 | 4AS | scf7180014970248_4u | 4U | 1151.84 | 0.00 × 10^+00^ | 98.93 | 649 | Lovegrove et al. 2013 |
| wheat | *TaGT43-2B* | HF913568.1 | 4AS | scf7180014970248_4u | 4U | 1130.20 | 0.00 × 10^+00^ | 98.17 | 644 | Lovegrove et al. 2013 |
| wheat | *TaGT43-2A* | HF913569.1 | 4AS | scf7180014970248_4u | 4U | 1115.77 | 0.00 × 10^+00^ | 97.71 | 641 | Lovegrove et al. 2013 |
| wheat | *TaGT43-4* | HM236487.1 | 7AL, 7BL, 7DL | scf7180014328570_7ul | 7UL | 1330.37 | 0 | 94.42 | 796 | Zeng et al. 2010 |
| wheat | *TaGT47-2B* | HF913570.1 | 3B | scf7180011400473_3u | 3U | 1011.17 | 0.00 × 10^+00^ | 96.57 | 592 | Lovegrove et al. 2013 |
| wheat | *TaGT47-2D* | HF913571.1 | 3AL | scf7180011400473_3u | 3U | 942.64 | 0.00 × 10^+00^ | 96.67 | 551 | Lovegrove et al. 2013 |
| wheat | *TaGT47-2A* | HF913572.1 | 3AL | scf7180011400473_3u | 3U | 949.86 | 0.00 × 10^+00^ | 95.92 | 564 | Lovegrove et al. 2013 |
| wheat | *TaGT47-12* | HM236486.1 | 3AL, 3B, 3DL | jcf7180007991296_6u | 6U | 598.20 | 4.61 × 10^-168^ | 98.55 | 339 | Zeng et al. 2010 |
| wheat | *TaGT47-13* | HM236485.1 | 3AL, 3B, 3DL | scf7180011400473_3u | 3U | 464.75 | 9.06 × 10^-128^ | 79.96 | 415 | Zeng et al. 2010 |
| wheat | *TaGT61-1* | FR873610.1 | 1BL | jcf7180008039709_6u | 6U | 1963.35 | 0.00 × 10^+00^ | 96.48 | 1151 | Anders et al. 2012 |
| wheat | *TaGT61-2* | FR846232.1 | 6AL | scf7180015018236_4u | 4U | 2282.55 | 0.00 × 10^+00^ | 96.87 | 1331 | Anders et al. 2012 |
| wheat | *TaGT75-1* | HM236488.1 | 2AL, 2BL, 2DL | scf7180031528207_2u | 2U | 1703.67 | 0.00 × 10^+00^ | 96.08 | 1006 | Zeng et al. 2010 |
| wheat | *TaGT75-4* | HM236489.1 | 4AL, 4BS, 4DS | jcf7180008104047_6u | 6U | 600.00 | 1.32 × 10^-168^ | 98.27 | 341 | Zeng et al. 2010 |
|  |  |  |  |  |  |  |  |  |  |  |
| wheat | *TaBAHD1A* | Traes_3AS_75E04A7F4** | 3AS | scf7180011408548_3u | 3U | 536.18 | 7.95 × 10^-179^ | 97.36 | 258 |  |
|  |  |  |  |  |  |  |  |  |  |  |

*: NCBI (https://www.ncbi.nlm.nih.gov/); **EnsemblPlants (<http://plants.ensembl.org/>)

Anders, N., Wilkinson, M.D., Lovegrove, A., [Freeman, J](http://apps.webofknowledge.com/DaisyOneClickSearch.do?product=WOS&search_mode=DaisyOneClickSearch&colName=WOS&SID=T1p2FchIeUoECnQrI9s&author_name=Freeman,%20J&dais_id=26056350&excludeEventConfig=ExcludeIfFromFullRecPage&cacheurlFromRightClick=no)., [Tryfona, T](http://apps.webofknowledge.com/DaisyOneClickSearch.do?product=WOS&search_mode=DaisyOneClickSearch&colName=WOS&SID=T1p2FchIeUoECnQrI9s&author_name=Tryfona,%20T&dais_id=80869080&excludeEventConfig=ExcludeIfFromFullRecPage&cacheurlFromRightClick=no)., [Pellny, T.K](http://apps.webofknowledge.com/DaisyOneClickSearch.do?product=WOS&search_mode=DaisyOneClickSearch&colName=WOS&SID=T1p2FchIeUoECnQrI9s&author_name=Pellny,%20TK&dais_id=61069410&excludeEventConfig=ExcludeIfFromFullRecPage&cacheurlFromRightClick=no)., et al. (2012). Glycosyl transferases in family 61 mediate arabinofuranosyl transfer onto xylan in grasses. [*PNAS*](javascript:;) 109, 989-993.

Lovegrove, A., Wilkinson, M.D., Freeman, J., [Pellny, TK](http://apps.webofknowledge.com/DaisyOneClickSearch.do?product=WOS&search_mode=DaisyOneClickSearch&colName=WOS&SID=T1p2FchIeUoECnQrI9s&author_name=Pellny,%20TK&dais_id=61069410&excludeEventConfig=ExcludeIfFromFullRecPage&cacheurlFromRightClick=no)., Tosi, P., Saulnier, L., et al. (2013). [RNA interference suppression of genes in glycosyl transferase families 43 and 47 in wheat starchy endosperm causes large decreases in arabinoxylan content.](http://apps.webofknowledge.com/full_record.do?product=WOS&search_mode=GeneralSearch&qid=3&SID=T1p2FchIeUoECnQrI9s&page=2&doc=16&cacheurlFromRightClick=no) [*Plant Physiology*](javascript:;) 163, 95-107.

Zeng, W., Jiang, N., Nadella, R., Killen, T.L., Nadella, V., Faik, A. (2010). A glucurono(arabino)xylan synthase complex from wheat contains members of the GT43, GT47, and GT75 families and functions cooperatively. *Plant Physiology* 154, 78–97.
